# Exploring the Contextual Sensitivity of Factors that Determine Cell-to-Cell Variability in Receptor-Mediated Apoptosis

**DOI:** 10.1371/journal.pcbi.1002482

**Published:** 2012-04-26

**Authors:** Suzanne Gaudet, Sabrina L. Spencer, William W. Chen, Peter K. Sorger

**Affiliations:** 1 Department of Cancer Biology and Center for Cancer Systems Biology, Dana-Farber Cancer Institute, Boston, Massachusetts, United States of America; 2 Department of Genetics, Harvard Medical School, Boston, Massachusetts, United States of America; 3 Center for Cell Decision Processes, Department of Systems Biology, Harvard Medical School, Boston, Massachusetts, United States of America; New York University, United States of America

## Abstract

Stochastic fluctuations in gene expression give rise to cell-to-cell variability in protein levels which can potentially cause variability in cellular phenotype. For TRAIL (TNF-related apoptosis-inducing ligand) variability manifests itself as dramatic differences in the time between ligand exposure and the sudden activation of the effector caspases that kill cells. However, the contribution of individual proteins to phenotypic variability has not been explored in detail. In this paper we use feature-based sensitivity analysis as a means to estimate the impact of variation in key apoptosis regulators on variability in the dynamics of cell death. We use Monte Carlo sampling from measured protein concentration distributions in combination with a previously validated ordinary differential equation model of apoptosis to simulate the dynamics of receptor-mediated apoptosis. We find that variation in the concentrations of some proteins matters much more than variation in others and that precisely which proteins matter depends both on the concentrations of other proteins and on whether correlations in protein levels are taken into account. A prediction from simulation that we confirm experimentally is that variability in fate is sensitive to even small increases in the levels of Bcl-2. We also show that sensitivity to Bcl-2 levels is itself sensitive to the levels of interacting proteins. The contextual dependency is implicit in the mathematical formulation of sensitivity, but our data show that it is also important for biologically relevant parameter values. Our work provides a conceptual and practical means to study and understand the impact of cell-to-cell variability in protein expression levels on cell fate using deterministic models and sampling from parameter distributions.

## Author Summary

Variability among members of a clonal cell population is increasingly recognized as a near-universal characteristic of prokaryotic and eukaryotic cells. Variability can arise from random fluctuations in the biochemical reactions that control gene transcription, protein synthesis or signal transduction networks. For variability in receptor-mediated signaling responses (in the current work, those activated by the death-inducing ligand TRAIL), we can often distinguish between the influence of stochastic processes that occur prior to ligand exposure and those that occur subsequently. One manifestation of prior variability is cell-to-cell differences in protein concentrations, and this paper uses a combination of modeling and experimentation to ask how these differences impact variability in phenotype, specifically with respect to the timing and probability of cell death. We find that fluctuations in multiple proteins contribute jointly to phenotypic variability, that the contributions of specific proteins to phenotypic variability are highly sensitive to the concentrations of other proteins, and that correlations in protein levels (detectable experimentally) also have a measurable impact on phenotype. Our work provides insight into the regulation of apoptosis and also represents a general approach for understanding cell-to-cell variability in signal transduction pathways.

## Introduction

Variability in the responses of tumor cells to biological stimuli is often ascribed to genetic differences. However, it has become increasingly clear that even genetically identical cells growing in a homogenous environment respond differently to ligands, drugs, or other stimuli. Non-genetic variability at the single-cell level has been demonstrated in the activation of immune responses [1], [2], [3], [4], viral infectivity [5], [6], [7], developmental fate [8], [9], [10], [11], antibiotic resistance [12], and sensitivity to therapeutic drugs [13], [14], [15]. Such variability can arise from relatively long-lasting “epigenetic” changes that have their origins in stable and heritable programs of gene expression [16] and can be sensitive to histone deactylase inhibitors that disrupt the histone code [14]. Substantial phenotypic variability also arises from fluctuation in the levels or activities of proteins (or other biomolecules) that control cell fate; the current paper is concerned with this type of variability.

Two sources of non-genetic variability can be distinguished. The first, often called “intrinsic noise”, arises when the copy number of molecules participating in a reaction under study is sufficiently small that probabilistic fluctuations in protein-protein interactions or biochemical reactions have observable effects [17]. Such processes are modeled using stochastic methods. The second source of variation, often called “extrinsic noise,” arises when protein concentrations in individual cells are high enough that single-cell reaction trajectories are well approximated by mass-action kinetics, but “external” or pre-existing cell-to-cell differences in the activities or concentrations of biomolecules have an effect [17]. With either intrinsic or extrinsic noise, phenotypes vary from one cell to the next but the processes that cause cells to differ are either part of or external to the biological process under study.

When clonal cell populations are treated with TNF-related apoptosis inducing ligand (TRAIL), their response is dramatically different from cell to cell: some cells die with 45 min, some die after as long as 12 hr, and some do not die at all [15], [18]. We have investigated the contributions of intrinsic and extrinsic noise to this variability by studying sister cells [15]. Were cell-to-cell variability to arise predominantly from intrinsic noise, we would expect sister cells to be no more correlated phenotypically than two cells selected at random from a population: intrinsic noise cannot be inherited. However, time-lapse microscopy has shown that the time and probability of TRAIL-induced cell death are highly correlated in newly born sister-cell pairs. The correlation in time of death between sister cells decays on a time scale of hours to days so that older sister cells are ultimately no more similar to each other than are pairs of cells selected at random from the population. Were cell-to-cell variability in phenotype to arise from differences in protein levels or activities, we would expect phenotypes to be transiently heritable (as observed with TRAIL) because binomial partitioning of cellular contents at division causes sisters to inherit similar numbers of high abundance biomolecules [19], [20]. Subsequent decorrelation in protein levels, and thus in time and probability of death, is also expected because fluctuations in protein synthesis and degradation (processes that exhibit significant intrinsic noise [6]) have an increasing impact as time progresses. The time required for sister cells to diverge and recapitulate the steady state distribution is known as the “remixing time”. Factors that determine remixing times are not fully understood [8], [9], [21] but translation rates are one contributor. Because cell-to-cell variability in responses to TRAIL can be ascribed primarily to differences in protein concentrations existing at the time of ligand addition and not to intrinsic noise in signal transduction reactions, deterministic mass-action modeling is appropriate [15]. Indeed, attempts to reproduce observed variability in cell death dynamics using conventional stochastic simulations have not succeeded, probably because proteins that regulate apoptosis are abundant [22].

**Figure 1:**
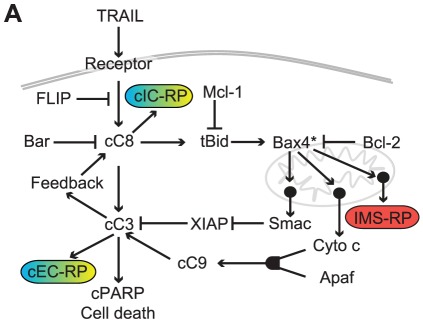
The TRAIL-induced signaling network. (**A**) Schematic diagram of the TRAIL-induced cell death signaling network including live-cell imaging reporters for MOMP, the inter-membrane space reporter protein (IMS-RP), and for initiator or effector caspase activity (IC-RP or EC-RP, respectively). The features *t_PARP_*, *f_PARP_*, and *t_switch_* can all be evaluated based on EC-RP dynamics and *t_MOMP_* can be measured in live cells using IMS-RP. IC-RP enables measurement of the threshold of cleaved initiator caspase substrate required for MOMP and of an initial rate of caspase activity (*k_IC_*). See also Figure 2 and Table Box 1 for description of how reporter dynamics were modeled and for precise definitions of features.

TRAIL-mediated apoptosis involves binding of TRAIL ligand to transmembrane DR4/5 receptors and consequent activation of effector caspases. To simulate these processes we have developed a series of mass-action models based on networks of ordinary differential equations (ODEs; referred to as *e*xtrinsic *a*poptosis *r*eaction *m*odels, or EARMs) that have been validated in single-cell studies using small molecule drugs, pathway-wide RNAi, and protein overexpression [18]. EARM describes the dynamics of death in single cells with good accuracy, particularly when cells are exposed to low-dose cycloheximide that blocks de novo protein synthesis (from the perspective of modeling, use of cycloheximide obviates the need to model TRAIL-induced transcription and translation and reduces the number of model parameters). Upon TRAIL stimulation, death-inducing signaling complexes (DISCs) assemble on the cytoplasmic tails of TRAIL-bound DR4/DR5 receptors, activating initiator pro-caspases-8 and -10 (hereafter referred to as caspase-8 or C8 for simplicity, Figure 1). Active caspase-8 directly cleaves effector pro-caspases-3 and -7 (hereafter simplified to caspase-3 or C3) but in most cell types, including those studied here, caspase-3 activity is held in check by XIAP until mitochondrial outer membrane permeabilization (MOMP) takes place. MOMP is controlled by members of the Bcl-2-family of proteins, which includes both positive and negative regulators. Active caspase-8 cleaves Bid into tBid which then induces a conformational change in Bax. Active Bax translocates to the mitochondria, where it (or its homolog Bak) multimerizes and form transmembrane pores. Pore assembly is antagonized by anti-apoptotic Bcl-2 proteins present in the cytosol and outer mitochondrial membrane. Only when levels of active Bax/Bak exceed those of inhibitory Bcl-2 proteins does pore formation begin and MOMP take place, releasing cytochrome *c* and Smac into the cytosol in a sudden, all-or-none process. Cytochrome *c* forms an apoptosome complex that also contains Apaf-1 and activates caspase-9, thereby creating an additional factor capable of processing pro-caspase-3. Smac binds to XIAP, which prevents XIAP from associating with active caspase-3, freeing caspase-3 so it can cleave substrates such as the inhibitor of caspase-activated DNase (ICAD) and poly (ADP-ribose) polymerase (PARP), and thereby promote fragmentation of the genome and proteome.

The current work aims to evaluate the impact of changes in the concentrations of apoptosis regulators on the dynamics of effector caspase activation in cells treated with TRAIL, particularly for changes that arise from natural variation in protein levels from one cell to the next. Because we observe such variation to be constant across a continuously growing cell population (that is, to be quasi-static) time-invariant distributions of initial protein concentrations can be used as inputs for ODE modeling. We measured protein concentration distributions in asynchronous cell populations using flow cytometry and microscopy and, when suitable reagents were available we also measured correlations in the concentrations of different proteins. Variability in the dynamics of apoptosis was then simulated by sampling from these distributions. In principle, we expect variation in the levels of some proteins to matter more than variation in others, and we show that this is indeed the case. We explore the impact of correlations in the levels of one or more model species and also ask how many species must be measured to accurately predict phenotype. Finally, we show that the phenotypic consequences of variation in particular proteins are affected by changes in the concentrations other apoptotic regulators, demonstrating contextual dependency in the contributions made by regulatory molecules to the timing and probability of cell death.

## Results

### Feature-based sensitivity analysis of cell death dynamics

Cell death is represented in EARM by caspase-mediated proteolysis of PARP to generate cleaved PARP (cPARP): previous studies have shown that HeLa cells are inviable when cPARP exceeds ∼10% of initial PARP levels ([cPARP]>0.10*[PARP]_0_) [23]. Under normal circumstances, cleavage of effector caspase substrates constitutes a “snap-action” switch that is well described by a sigmoidal Boltzmann trajectory in which an extended delay is followed by sudden and complete PARP proteolysis [18], [24]. The delay time is long (45 min to 12 hr), varies from cell to cell, and increases as the dose of TRAIL decreases. In contrast, the rate of PARP cleavage is rapid once begun, and dose-invariant (the time between the first detectable cleavage of PARP and its completion is typically 20–25 min). However, RNAi-mediated depletion of regulatory proteins such as XIAP or treatment of cells with proteasome inhibitors such as MG132 changes the dynamics so that PARP is cleaved more slowly and may not go to completion. We have previously argued that these qualitative changes create a pathological state in which caspase-activated DNase is active, genomic DNA damaged, but some damaged cells do not die [18], [23]. Such cells have been proposed to play a role in tumor initiation [25], [26], [27], [28]. Thus there is a fundamental difference between natural variation in the timing of apoptosis and the breakdown associated with slow and incomplete execution of the apoptosis program.

The impact of changes in the initial protein concentrations or other parameters on model output is determined using sensitivity analysis. For extrinsic apoptosis we can distinguish between normal and pathological behaviors using four features of cPARP and cytosolic Smac trajectories: 1) *t_PARP_*, the time between ligand exposure and 50% PARP cleavage (i.e. time of cell death), 2) *t_MOMP_*, the time between ligand exposure and MOMP (defined experimentally as the first image in which a fluorescent MOMP reporter appears diffuse in the cytoplasm and in the model as the time at which 50% of Smac has translocated to the cytosolic compartment), 3) *t_switch_*, the time between the start and finish of PARP cleavage (a measure of the rate of PARP cleavage), and 4) *f_PARP_*, the fraction of PARP cleaved at the end of the simulation or experiment (a measure of the completeness of apoptosis). In unperturbed HeLa cells exposed to 50 ng/ml TRAIL and 2.5 µg/ml cycloheximide, *t_PARP_* and *t_MOMP_* varied from 2–6 hr, *t_PARP_* occurred ∼10 min after *t_MOMP_* (at a single cell level), *t_switch_* was ∼25 min, and *f_PARP_* was ∼1.0 [23], [29].

The sensitivities of these features to changes in parameter values are related to but distinct from conventional sensitivities. Analytical expressions for feature sensitivities are described in Box 1 but the calculations in this paper actually involve numerical methods that account for complex non-local effects (see Text S2 and Box 2 for further details). In the numerical approach, sensitivities are calculated by Monte Carlo sampling of initial protein concentrations thereby repeatedly evaluating the local slope of the response curve for a feature. We evaluated feature sensitivities with respect to 16 non-zero initial protein concentrations individually by simulating PARP cleavage and Smac translocation dynamics in cells exposed to 50 ng/ml TRAIL while sampling uniformly in the exponent over a range of 10^2^–10^7^ proteins per cell (all other parameters remained at their nominal values). This range of concentrations reflects the range of possible concentrations for proteins in a mammalian cell [30], [31]; for apoptotic regulators it is a reasonable approximation to the concentration range achieved by protein overexpression or RNAi-mediated protein depletion. EARM has been validated using such methods and it performs well under these conditions [15], [23], [29], although most analysis has been performed for protein levels present in unperturbed HeLa cells.

**Box 1. Feature-based sensitivity**

**Figure 2:**
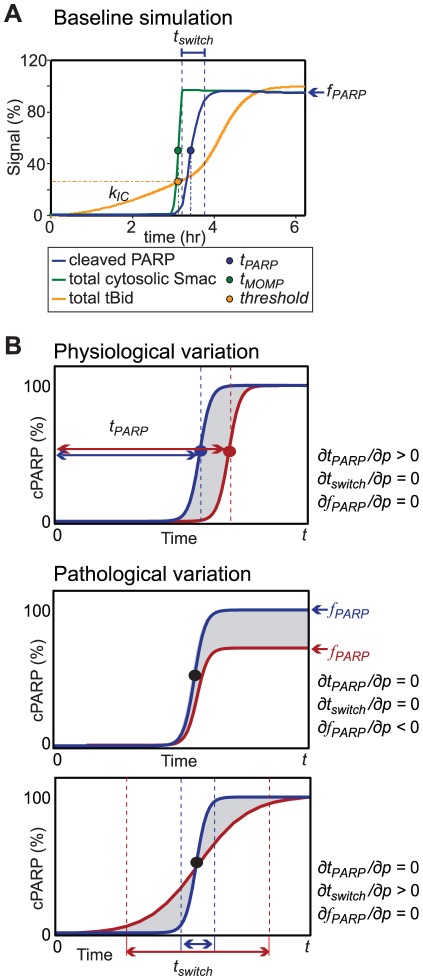
Feature-based description and sensitivity of apoptosis dynamics. (**A**) Plot showing the simulated time courses of cleaved PARP (blue; an effector caspase substrate), total cytosolic Smac (green) and total cleaved Bid (tBid, yellow; an initiator caspase substrate). Cleaved PARP corresponds to model species 23. Total cytosolic Smac is the sum of Smac_r (species 47), Smac (species 45) and Smac:XIAP (species 57). Total cleaved Bid is the sum of tBid (species 26), tBid:Bax (species 28), and tBid:Mcl1 (species 30). The four model features under investigation are indicated (*t_MOMP_*, *t_PARP_*, *f_PARP_*, and *t_switch_*), as well as two features used to classify proteins in Figures 4 and 7 (the initiator caspase rate, *k_IC_*, and *threshold*). (**B**) Schematic representations of three classes of changes in the cPARP trajectory and the corresponding time-integrated value of the parameter sensitivity (gray). Changes that are quantitatively similar in terms of conventional sensitivity are distinct by feature sensitivity. The same qualitative distinctions apply to sensitivities calculated in the limit of an infinitesimal change in parameter values. Therefore the curves in panel B are related to feature sensitivities (and to Equation 1) as shown by the expressions on the right.

Parameters in mass-action biochemical models include initial protein concentrations, and kinetic parameters such as association, dissociation, and catalytic rate constants, Hill coefficients etc. The impact of changes in parameter values on model outputs, typically dynamical variables such as the concentrations or activities of proteins over time, is calculated using sensitivity analysis. In EARM, the dynamical variable reporting on MOMP is the level of cytosolic Smac and the variable reporting on effector caspase activity is the level of cleaved PARP (cPARP) (Figure 2A). The conventional sensitivities of these variables are the normalized changes at a particular point in time arising from an infinitesimal change in a parameter value. Often, these partial derivatives are integrated over time (Figure 2B, gray areas).

If we consider the impact of RNAi and naturally occurring cell-to-cell variability on the trajectories of cytosolic Smac or cPARP we realize that conventional sensitivity values are not particularly informative: variation in the timing of cPARP accumulation is normal (Figure 2B, top panel) while incomplete or abnormally slow PARP cleavage is pathological (Figure 2B, center and bottom panels respectively), but the time integrated sensitivities for all three scenarios are similar (approximated by the gray areas between the curves). Physiological and pathological changes in cPARP dynamics can be discriminated using *features* of the trajectories such as the time at which PARP is 50% cleaved (*t_PARP_*), the time required for cPARP to go from 10% to 90% cleaved (*t_switch_*) or its final fraction relative to [PARP]_0_ (*f_PARP_*) (Figure 2A; mathematical definitions in Table 1). As shown in this paper, feature-based sensitivities are more effective than conventional sensitivity in analyzing apoptotic regulators because they report on biologically meaningful variation. The concept of feature-based sensitivity can also be reformulated for different types of trajectories in any dynamical system and these sensitivities can be computed locally (e.g. Figure S1) or as part of global sensitivity analysis (see for example algorithms in ref. [42], [43], [44] and reviewed in ref. [45]).

**Figure 3:**
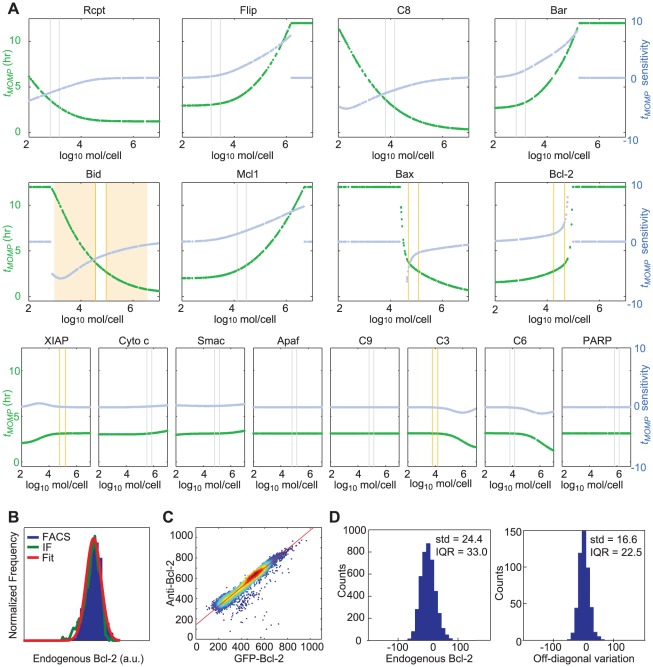
Sensitivity of *t_MOMP_* to changes in protein initial concentrations and measurements of protein variance and co-variance in HeLa cells. (**A**) Scatter plots show the simulated relationship between initial protein concentration and *t_MOMP_* (green) or numerically calculated *t_MOMP_* sensitivity (blue; *t_MOMP_* sensitivity is unitless and is calculated using finite-difference approximations of the derivatives, or slopes, of the green curves) following TRAIL addition, for the indicated proteins. The initial concentration for the indicated protein was uniformly sampled in the exponent for values between 10^2^ to 10^7^ proteins per cell while all other initial protein concentrations and rate constants were set at their default value. Vertical bars represent the 5^th^ and 95^th^ percentiles of the measured (orange, see panel B–D and Table S2) or assumed (gray) distributions in endogenous protein concentrations for untreated HeLa cells. Shaded regions in the plot for Bid show an example of concentration ranges that were attained experimentally using RNAi knockdown and GFP-fusion protein overexpression [15], [23], [29]. (**B**) Overlays of endogenous Bcl-2 concentration distributions in untreated HeLa cells as measured by flow cytometry (FACS, blue), or immunofluorescence (IF, green). The FACS data are well fit by a log-normal distribution (Fit, red); a.u., arbitrary units. (**C**) Scatter plot of anti-Bcl-2 vs. GFP-Bcl-2 signal in GFP-Bcl-2-transfected HeLa cells measured by 2-color flow cytometry. (**D**) Histograms of the endogenous Bcl-2 concentration distribution in wildtype HeLa cells measured with an anti-Bcl-2 antibody (left) and of the off-diagonal noise distribution for the scatter plot in (B) (right). Both distributions are for mean-centered data to allow comparison of variability; std is the standard deviation and IQR is the interquartile range.

The sensitivity of time-based features such as *t_PARP_* or *t_MOMP_* is approximated by:
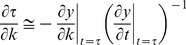
(1)where *k* is a model parameter, *τ* represents the time-based feature of interest (*t_PARP_*, *t_MOMP_*) and *y* is the dynamical variable that governs the feature (cPARP and cytosolic Smac, for *t_PARP_* and *t_MOMP_* respectively; the derivation of Equation 1 is described in Text S2). Thus, feature sensitivity is approximately equal to the conventional sensitivity 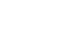 divided by the slope of the trajectory 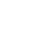 evaluated at the appropriate time point for the feature (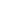; e.g. when PARP is 50% cleaved for *t_PARP_* or when Smac is 50% cytosolic for *t_MOMP_*). Equation 1 also has an appealing geometric interpretation when we graph *y* as a function of time, as we illustrate in Text S2. Equation 1 is valid in the ideal case where MOMP and PARP cleavage are observed in every simulation, however when simulations are performed for finite time intervals (and not all cells die in certain regions of *k*) the formula breaks down (Figure S1 in Text S1). A practical alternative is to use numerical methods for computing *t_MOMP_* and *t_PARP_* (see main text and Figures 3A and S2, S3, S4).

Both *t_switch_* and *f_PARP_* can be defined by expressions based on evaluating the cPARP dynamic variable at specific time points, and therefore exact analytical expressions can be derived for their sensitivities to changes in parameter values; these are listed in Table 1.

**Table 1:** Mathematical definitions of features of the apoptosis process modeled in EARM and their analytical expressions for parameter sensitivity.

Feature	Analytical expression for feature sensitivity
*t_MOMP_* is *t* at which: 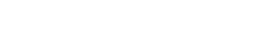	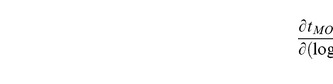
*t_PARP_* is *t* at which: 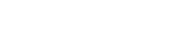	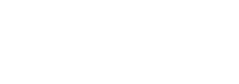
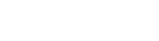	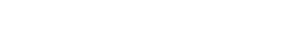
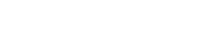	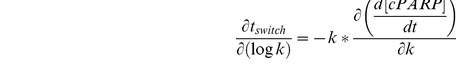

**Box 2. Variance in system behavior relates to variance and co-variance in parameter values**

Although it is conventional to emphasize individual determinants of cellular phenotype, natural variation in TRAIL-mediated cell death is regulated by multiple factors in surprisingly subtle ways ([15], [23], [29], [34]; and this work). We can understand why this is true by deriving an approximate relationship between the variance of a feature and changes in the level of a particular parameter; in the current work we are particularly concerned with the impact of naturally occurring fluctuations in initial protein concentrations. Variance of feature *q* (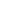) is approximately:
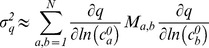
(2)where the indices *a, b* refer to pairs of *N* concentration parameters, 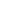 and 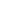 are their initial concentrations (in log units) and 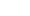 is the covariance matrix of concentrations (see Supplemental Text S1 for the derivation). If protein levels are assumed to be uncorrelated then off-diagonal terms are zero and the entire expression is reduced to a sum of squared terms over all proteins having non-zero initial concentrations:
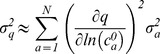
(3)From Equation 3 we see that variances in protein concentrations contribute only positively and additively to variances in features. A caveat to Equations 2 and 3 is that sensitivities are local and the approximations do not account for changes in sensitivity as parameters vary; these effects (which can be large in some cases) are most easily estimated with numerical methods such as Monte Carlo sampling of parameter distributions (as in Figure 3). Nevertheless, Equation 2 would provide an extensible approach to approximate the impact of covariance of sets of three or more parameters.

We can also use Equation 2 to understand the possible phenotypic consequences of measured or postulated correlations in protein concentrations. The right-hand side of Equation 2 is a product of three terms comprising two feature sensitivities and co-variation between two concentrations. Sensitivities can be positive or negative (“anti-apoptotic” or “pro-apoptotic” if considering *t_PARP_* or *t_MOMP_*, for example) and co-variation between two protein concentrations can also be positive or negative, although positive co-variation is expected [35] in the absence of a specific regulatory mechanism to enforce negative co-variation (e.g. by a ubiquitin ligase and its target). Considering the sign of individual terms, we arrive at four scenarios (Table 2). In scenario 1, in which sensitivities to variation in two parameters have opposite signs (one positive and one negative) and covariance is positive, variation in features controlled by the parameters will decrease, precisely what we observe for XIAP and Smac (Figure 5B). Conversely, positive covariance for two parameters having the same influence on a feature (either negative or positive) will increase variance in the feature (scenario 2), which is what is observed for the pro-apoptotic proteins Apaf1 and Smac (Figure 5B). Scenarios 3 and 4 are the converse, and pertain to situations where covariance is negative.

**Table 2:** Impact of co-varying protein concentrations on variation in features.

Scenario	Sensitivity of A, B^a^	Co-variation of A,B	Impact on variance in feature
1	**+,− or −,+**	**+**	**−**
2	**+,+ or −,−**	**+**	**+**
3	**+,− or −,+**	**−**	**+**
4	**+,+ or −,−**	**−**	**−**

Notes:

a “A” and “B” refer to two parameters for which a feature sensitivity has been computed.

The sensitivity of model features to changes in protein levels broadly conformed to expectation: for *t_MOMP_*, higher levels of pro-apoptotic proteins lying upstream of pore formation decreased its value (Receptor, caspase-8, Bid, and Bax; Figure 3A, green curves show responses, blue curves show sensitivities) whereas higher levels of upstream anti-apoptotic proteins (FLIP, Bar, Mcl-1, Bcl-2; Figure 3A) increased its value. In contrast, changes in the levels of downstream proteins had little effect (e.g. caspase-3, caspase-9, Apaf1; Figure 3A, bottom row). The sensitivities of other features are shown in Figures S2, S3, S4 in Text S1. They reveal that different features exhibit different sensitivity with respect to protein initial concentrations. These sensitivities varied considerably in magnitude with position in parameter space: over some concentration ranges, small changes in the levels of proteins such as FLIP, Mcl-1 or Bcl-2 had a large impact on model output but over other ranges the impact of small changes was minimal (Figure 3A). For example, when [Bcl-2] lay between 10^4^ and 10^5^ molecules per cell, *t_MOMP_* changed rapidly whereas with [Bcl-2] between 10^2^ and 10^4^ molecules per cell, *t_MOMP_* changed very little. Because sensitivity is a local property of a model, this result is expected from a mathematical perspective but is often overlooked from a biological perspective.

To estimate mean concentrations of apoptotic regulators in HeLa cells, we used calibrated immunoblotting and recombinant protein standards (Figure S5 in Text S1); to estimate variation in protein levels from one cell to the next we used flow cytometry ([15]; Figures S6, S7, S8 in Text S1). The selectivity of antibodies was validated using siRNA-mediated protein knockdown and/or protein over-expression (Figure S7 in Text S1). Bcl-2, for which good antibodies are available, was assayed using both immunofluorescence microscopy and flow cytometry and we observed excellent agreement between the two types of measurement (Figure 3B). Both measurements rely on immunodetection and we wanted to exclude the possibility that variability in antibody-antigen binding might have a significant impact on the measured distributions. We therefore transfected cells with a construct expressing GFP-Bcl-2 and quantified the intensities of GFP and of anti-Bcl-2 immunofluorescence in the same cells (Figure 3C). In a two-dimensional scatter plot of these measurements, variation along the diagonal represents real cell-to-cell differences in Bcl-2 concentration whereas off-diagonal variation represents differences between antibody-based and GFP-based estimates of Bcl-2 abundance. Off-diagonal variation can arise from antibody binding, instrument error, the presence of some immature and non-fluorescent GFP-Bcl-2 molecules, or variability in levels of endogenous Bcl-2. Off-diagonal variation therefore represents an upper-bound estimate of measurement error arising from antibody binding and detection. We observed that off-diagonal variation was significantly smaller than natural cell-to-cell variation in endogenous Bcl-2 levels, suggesting that estimates of variability in Bcl-2 levels do indeed reflect real differences in protein abundance from cell to cell (Figure 3D).

Across a set of five proteins for which we could demonstrate antibody selectivity (only a subset of commercially available antibodies are suitable), measured distributions of protein abundance were unimodal and long-tailed as has been observed previously in mammalian cells [21], [32]. All were well fit by log-normal distributions, although we cannot exclude the possibility that gamma distributions or other long-tailed distributions are also appropriate representations of the data (Figure S8 in Text S1). The coefficients of variation (CV; standard deviation divided by mean) were between 0.43 and 0.47 but some of this variation is expected to arise from differences in cell size. We therefore selected cells with similar forward and side scatter measurements, which reduced CVs to 0.28±0.02 to 0.30±0.03 depending on the protein. Given the relatively narrow range of values for the CV, it seemed reasonable to assume similar variance for those proteins we could not assay experimentally. We therefore set CV = 0.25 for proteins whose distributions were assumed rather than measured (to err on the conservative side). In Figure 3A we relate the sensitivity of *t_MOMP_* to endogenous protein concentrations in HeLa cells by positioning double vertical bars at the 5^th^ and 95^th^ percentiles of the protein concentration distributions (see also Figures S1, S2, S3, S4 for other features). In general, data and simulations predicted HeLa cells to be more sensitive to increases than to decreases in the levels of anti-apoptotic proteins across the endogenous range; the opposite is true for pro-apoptotic proteins. The endogenous distributions of Bcl-2 and Bax were particularly interesting because they suggested that HeLa cells lie close to a region of parameter space in which small changes are expected to have a large impact on *t_MOMP_*, a finding we analyze in greater detail below.

### Variability in some but not all protein concentrations changes death dynamics

**Figure 4:**
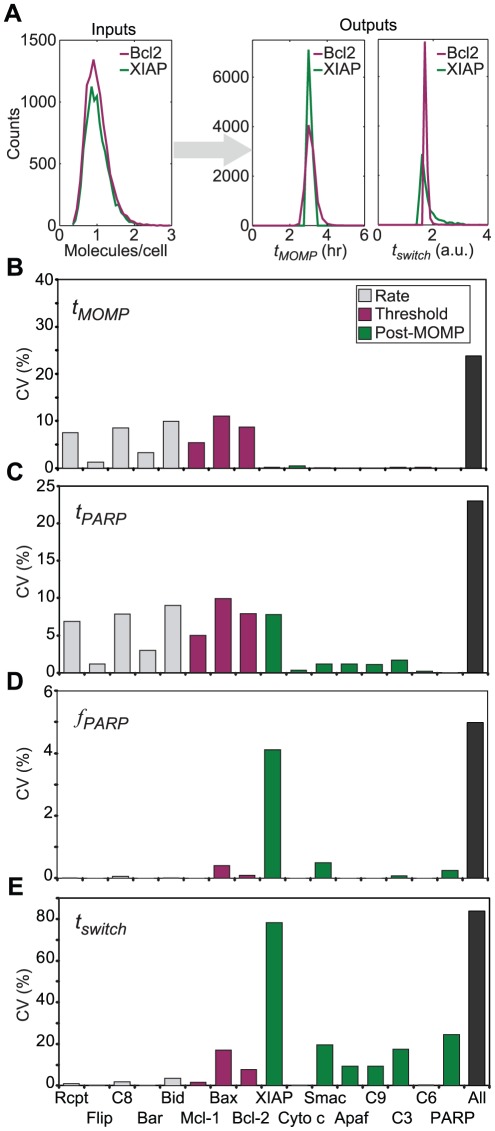
The impact of variability in protein initial concentrations is feature-specific. (**A**) Histograms showing the distributions of initial concentrations of Bcl-2 and XIAP used as inputs to the model (left) and the model output distributions for *t_MOMP_* and *t_switch_* (right). Input distributions were generated by sampling 10,000 times from a log-normal distribution parameterized with measured or assumed mean and CV as listed in Table S2 in Text S3. Output distributions were calculated from10^4^ simulations where the initial concentration of the indicated protein was sampled from the distributions shown on the left; all others protein concentrations were set to their default value (Table S2 in Text S3). (**B–E**) Bar graph showing the coefficients of variation (CV) obtained for model output distributions of *t_MOMP_* (**B**), *t_PARP_* (**C**), *f_PARP_* (**D**), and *t_switch_* (**E**) from series of 10^4^ simulations where the indicated protein initial concentration is sampled from a log-normal distribution and all other concentrations set to their default value (Table S2 in Text S3). Proteins were classified as affecting the pre-MOMP rate of initiator caspase activity (Rate; gray), the MOMP threshold (Threshold; purple) or post-MOMP processes (Post-MOMP; green) based on their position in the TRAIL-induced signaling network (Figure 1). In panels B–E, the black bar (“All”) indicates the variability observed in a series of 10^4^ simulations where all non-zero initial conditions were independently sampled from log-normal protein distributions using the measured CV where available or else CV = 0.25 (as listed in Table S2 in Text S3).

**Figure 5:**
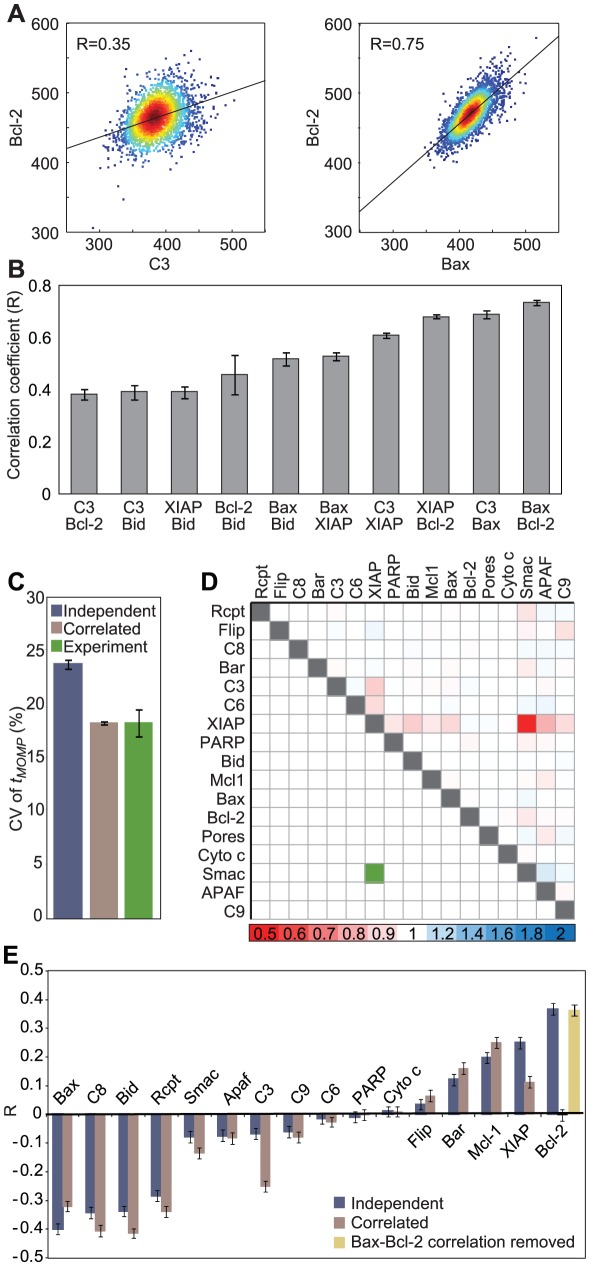
Correlations in protein levels affect the distribution of death times and the rank ordering of the most sensitive species. (**A**) Scatter plots showing joint measurements of the levels of pairs of proteins in a population of HeLa cells by flow cytometry (least correlated pair, Bcl-2 and caspase-3 (C3), left; most correlated pair, Bcl-2 and Bax, right). (**B**) Bar graph showing the measured Pearson correlation coefficients (calculated by linear regression) in the level of ten protein pairs. Error bars show the standard error of the mean. For all protein pairs, measurements were on cells that were size-selected by stringent gating for forward and side scatter. (**C**) Bar graph of the CVs of *t_MOMP_* distributions measured by monitoring IMS-RP translocation via time-lapse microscopy in HeLa cells treated with 50 ng/ml TRAIL with 2.5 µg/ml cycloheximide (green) or obtained from 10^4^ simulations using independent sampling of all initial conditions (independent, blue), or sampling from the experimentally determined joint distributions for Bax, Bcl-2, Bid, caspase-3 and XIAP (allowing all other proteins to vary independently; correlated, brown). Error bars represent standard deviations obtained by bootstrapping (n = 1000). Sampling for joint distributions reduced the predicted variability in *t_PARP_* from a CV = 0.23 to CV = 0.19, a statistically significant improvement in the match to experimental data: an Ansari-Bradley test for equal variability on median-corrected data yielded p = 0.006 for experimental data vs. independent sampling simulation, and p = 0.137 for experimental data vs. sampling with correlated distributions, rejecting the equal variance hypothesis only for data vs. independent sampling. (**D**) Heat map showing the effect of pairwise correlations on *t_PARP_* variability. Above the diagonal (gray), color indicates the ratio of the CV of *t_PARP_* for 10^4^ simulations with sampling from correlated vs. independent distributions for the indicated pair (all other proteins were sampled independently from log-normal distributions parameterized as in Table S2 in Text S3). Below the diagonal, the most significant p-value from a two-sample Kolmogov-Smirnov test is indicated in green (p = 0.04). (**E**) Bar graph of the Pearson correlation coefficients (R-values) of *t*_PARP_ with the indicated protein initial concentration for simulations sampling from fully independent distributions (blue), from joint distribution for Bax, Bcl-2, Bid, caspase-3 and XIAP (brown), or from a joint distribution for the same five proteins where the Bcl-2-Bax correlation was set to zero (yellow bar). Scatter plots of *t_PARP_* as a function of initial protein levels for the same simulation sets are presented in Figure S9 in Text S1.

To compute the impact of natural variation in protein levels on *t_MOMP_*, *t_PARP_*, *f_PARP_*, and *t_switch_*, we used Monte Carlo methods that sample from log-normal distributions of initial protein concentrations (based on measured or assumed values for the mean and variance; Figure 4A). The predicted distributions of all four features were unimodal regardless of whether we varied each protein concentration individually (with all others fixed at their nominal values; Table S2 in Text S3) or varied all proteins simultaneously (while sampling independently; Figure 4B–E). In no case did natural variation in the concentration of a single protein generate as much variation in *t_MOMP_* as simultaneous variation in all proteins (CV = 0.11 for *t_MOMP_* when Bax alone was varied as compared to CV = 0.24 for *t_MOMP_* when all proteins were varied independently). Results for *t_PARP_* were similar except that variation in XIAP levels (and to a lesser extent, variation in other proteins downstream of MOMP) also had an impact (Figure 5C). This is expected since XIAP is a direct negative regulator of caspase-3, and caspase-3 is the enzyme that cleaves PARP. From these data we conclude that experimentally observed variation in the time of MOMP or PARP cleavage is controlled in a multi-factorial manner and that total phenotypic variation cannot be explained by measured variation in any single protein concentration. A mathematical explanation of this effect is presented in Box 2.

In contrast, variability in *f_PARP_* was dominated by variability in the levels of XIAP, and the impact of varying XIAP alone was almost as great as that of varying all proteins simultaneously (Box 2, Equation 3 shows that it cannot be greater; Figure 5E). The situation was similar for *t_switch_*, except that factors downstream of MOMP also had an effect on this feature. These observations help to explain previous RNAi and over-expression data showing that forced changes in the levels of proteins that impact *f_PARP_* also affect *t_switch_*, and that both features are particularly sensitive to changes in the levels or activity of XIAP [18], [23], [33]. Indeed, the most potent way to reduce the efficiency of apoptosis experimentally in HeLa cells (i.e. increase *t_switch_* or reduce *f_PARP_*) and generate “half-dead” cells, appears to be to interfere with the levels or activity of XIAP [23]; the same is true in HCT116 human colon carcinoma cells [34]. Taken together, these results make the point that the “robustness” of a cell to variation in any single parameter is strongly dependent on the feature being evaluated: *t_MOMP_* is robust to variation in [XIAP] but *t_switch_* and *f_PARP_* are particularly sensitive to it. We find that virtually all of the proteins in the model are determining factors (sensitive parameters) for at least one physiologically important variable.

### Impact of co-variance in protein levels

Correlation in the levels of regulatory proteins is expected to alter the relationship between variability in protein concentration and variability in phenotype (Box 2, Equation 2). Using two-color flow cytometry, we measured correlations in the concentrations of Bax, Bcl-2, Bid, caspase-3 and XIAP across all ten pairwise combinations (suitable antibodies pairs were not available for other regulatory proteins). Gating on forward and side scatter was used to select for cells of similar size since positive correlation is expected simply based on cell volume. With stringent gating, we observed positive linear correlation coefficients that ranged from R∼0.4 for caspase-3 and Bcl-2 to R∼0.7 for Bax and Bcl-2 (Figure 5A–B). No negative correlations were observed, consistent with results from bacteria showing that extrinsic noise is expected to cause all protein levels to be positively correlated unless they are specifically counter-regulated [35].

To determine the impact of correlation in protein expression on model features we constructed a five-dimensional joint distribution based on pair-wise measurements and performed Monte Carlo sampling. Protein pairs whose co-variance is unknown were assumed to be uncorrelated. Including the measured correlations in initial protein concentrations reduced the predicted variability in *t_PARP_* from a CV = 0.23 to CV = 0.19, a statistically significant improvement in the match to experimental data (for which CV = 0.18; Figure 5C). We conclude that measured co-variation in protein levels has a significant impact on variability in the timing of death.

To investigate the impact of correlations in protein concentrations in cases in which experimental data could not be collected, we performed simulations considering each protein pair and assuming either independent distributions or a single joint pairwise distribution with R = 0.7 (the highest correlation observed experimentally; Figure 5B). All other proteins were sampled independently from their respective log-normal distributions. *t_PARP_* is the feature whose variance was affected by the greatest number of parameters (Figure 4B–E) and we therefore focused on it. For each pair of proteins in the model, we computed the ratio between the variance in *t_PARP_* expected under assumptions of independence or positive correlation (Figure 5D). In many cases effects were relatively modest. The largest single difference involved the anti-apoptotic XIAP protein and its pro-apoptotic binding partner Smac whose assumed correlation reduced dispersion in *t_PARP_* two-fold. This represents one example of a general phenomenon: positive correlations in the concentrations of pairs of proteins having opposing roles in apoptosis reduced the spread in death times (Figure 5D, red shading; see Box 2 for further explanation).

Although correlations in protein levels had a modest impact on variability in *t_PARP_*, it significantly altered the phenotypic consequences of variation in individual proteins. This can be seen by sampling from the experimentally determined joint distributions for Bax, Bcl-2, Bid, caspase-3 and XIAP (allowing all other proteins to vary independently) and using the Pearson correlation coefficient (*R*) to score the relationship between *t_PARP_* and the initial concentration of each model species (Figure 5E and Figure S9 in Text S1; similar results were obtained using Spearman's rank correlation coefficients, not shown). As expected, the *R* value for pro-apoptotic proteins was negative and for anti-apoptotic proteins it was positive (see also Figure S10 in Text S1 for an analysis yielding similar results using the slope of the regression line). Unexpectedly, Bcl-2 had virtually no correlation with *t_PARP_* (*R* = 0.002, Figure 5E) when concentrations were sampled from joint distributions even though it was significantly correlated when protein levels were assumed to be independent (*R* = 0.37). To try to explain this, we removed only the correlation between Bcl-2 and Bax from the joint distribution and re-computed *R* values for *t_PARP_*. This restored the impact of variance in Bcl-2 on *t_PARP_*, demonstrating the contextual dependency of feature sensitivities (Figure 5E). At the mechanistic level, this result can be explained by the fact that cells that have higher Bcl-2 levels (which should cause them to die later, on average) also have more Bax (causing them to die earlier, on average) because of positive correlation between Bcl-2 and Bax concentrations. Thus, the correlation between Bax and Bcl-2 dampens the variability in the [Bcl-2]_0_:[Bax]_0_ ratio and masks the impact of Bcl-2 on *t_PARP_*. More generally, correlations in protein concentrations can substantially alter individual parameter sensitivities and such effects could be strong enough to alter apparent mechanistic relationships between proteins and phenotypes. Conceivably, these effects could also be sufficient to mask the impact of forced changes in protein expression induced by RNAi or overexpression.

### Accurate prediction of time-to-death requires knowledge of many protein levels

**Figure 6:**
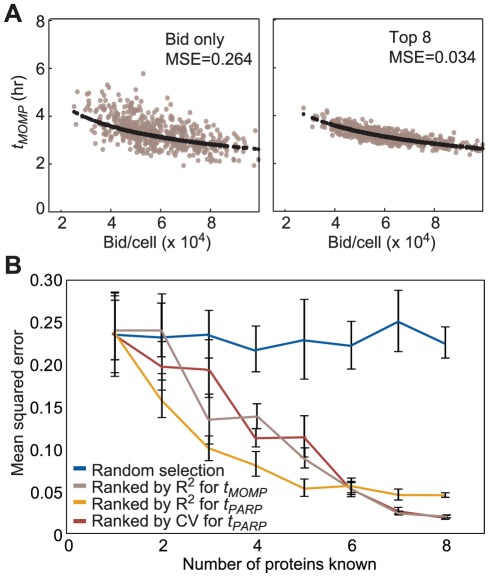
Predictability of death time is improved by knowledge of key protein concentrations. (**A**) Scatter plot of predicted *t_MOMP_* as a function of Bid initial concentration when no other initial protein concentrations are known (left) or when the initial concentrations of the next seven most influential proteins as ranked by R^2^ of *t_MOMP_* are also known with precision within ±12.5% (right). Simulations shown were selected from a series of 10^5^ simulations sampling from a joint distribution for Bax, Bcl-2, Bid, caspase-3 and XIAP (as measured) and independently for all other proteins with non-zero initial concentration. To mimic knowledge of a protein concentration, simulations were randomly selected from those with an initial concentration of mean value ±12.5% for this protein. Black points represent the predicted death times given perfect knowledge of the concentrations of all model species. MSE is the mean squared error relative to perfect knowledge (black points). (**B**) Graph of the mean squared error in *t_MOMP_* (relative to perfect knowledge, black points in (A)) as a function of the number of proteins whose concentration is “known”; values are the averages from different runs and error bars represent the standard deviations (n = 10). “Known” proteins were added either randomly (blue), by high-to-low R2 for *t_MOMP_* (gray; Figure S10) or *t_PARP_* (yellow; Figure 5E), or by high-to-low CV for *t_PARP_* (brown; Figure 4C).

To determine the practical consequences of multi-factorial control over *t_PARP_*, we asked how many measurements are required to accurately predict time of death in a single cell. We performed 10^5^ simulations of PARP cleavage in cells treated with 50 ng/ml TRAIL and low-dose cycloheximide assuming Bax, Bcl-2, Bid, caspase-3 and XIAP concentrations to co-vary and all other proteins to vary independently. We modeled the process of measuring one to eight proteins at a single-cell level assuming experimental error of ±12.5% and then computed how accurately *t_MOMP_* could be determined. In actual microscopy experiments *t_MOMP_* is typically sampled at 3 min intervals, resulting in a mean squared error (MSE) of ∼0.03. When we assumed knowledge only of [Bid]_0_, the MSE in predicting *t_MOMP_* was 0.26, a poor estimate given experimental error (reflected in the scatter of points around the trend line in Figure 6A, left). Next, we prioritized measurements by ranking them based on their contributions to variation in *t_PARP_* (as judged by CV or *R^2^* values with respect to *t_PARP_* as shown in Figures 4C and 5E) or to variation in *t_MOMP_* (as judged by *R^2^* values with respect to *t_MOMP_*, as shown in Figure S10 in Text S1). We observed that ability to predict *t_MOMP_* increased progressively (Figure 6B and Figure S11 in Text S1) and that knowledge of the seven or eight most sensitive protein concentrations was necessary to achieve an MSE approaching experimental error (i.e. 0.03; Figure 6A–B). In contrast, selecting proteins for measurement at random from the full set of 16 species having non-zero initial concentrations was ineffective in reducing the MSE. We conclude that accurate prediction of time of death from initial protein concentrations requires data on many proteins concentrations even in the best case. Single-cell measurement of 30 or more protein levels is now possible by mass cytometry [36], but these measurements destroy cells and it is therefore impossible to use the method to link multiplex measurement of protein concentration to events, such as cell death, that occur many hours later. In this sense, identifying the factors that determine the time of death of a single cell is not yet achievable experimentally, even though determining distributions of death times is straightforward.

### Bcl-2 over-expression shifts cells to a region of variable cell fate where Bax levels become the primary determinant of fate

**Figure 7:**
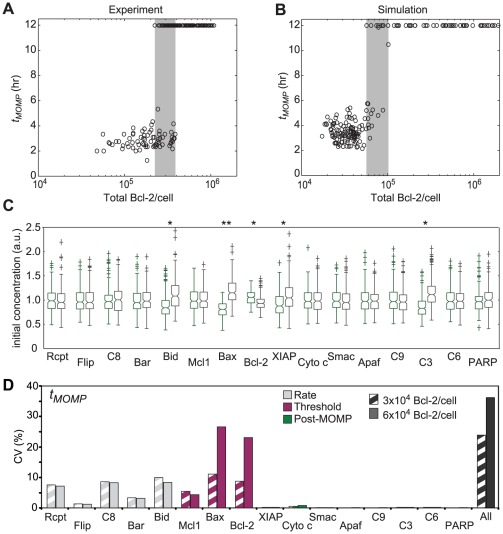
Overexpression of Bcl-2 in HeLa cells shows a region of variable fate before a threshold is reached where all cells survive. (**A–B**) Scatter plots showing the relationship between *t_MOMP_* and total Bcl-2 amount as measured in HeLa cells treated with 50 ng/ml TRAIL and 2.5 µg/ml cycloheximide (left) or simulated in EARM1.3, sampling linearly in the exponent for GFP-Bcl-2 levels and from a joint distribution for Bax, Bcl-2, Bid, caspase-3 and XIAP and independently for all other non-zero initial protein concentrations (right). Quantitative immunoblotting (Figure S5 in Text S1) and single-cell fluorescence quantification were combined to derive the absolute levels of GFP-Bcl-2 for each cell, to which the average endogenous Bcl-2 amount (30,000 molecules/cell; experimentally unobservable) was added to convert the *x*-axis to units of total Bcl-2 molecules per cell. Cells that did not undergo MOMP by 12 hr were assumed to have survived. (**C**) Boxplots of initial protein concentration distributions for surviving (green) or dying (gray) simulated cells selected for having a range of total Bcl-2 expression where ∼50% died (∼53,000–57,000 molecules/cell). Box edges show the 25^th^ and 75^th^ percentiles, notches show the 95% confidence interval for the median (horizontal line), and whiskers extend to the most extreme data points that are not considered outliers. Asterisks indicate proteins for which the surviving and dying simulated cells show significantly different medians for initial concentration (p<0.05), double asterisks mark the distributions for [Bax]_0_ which have the most significant difference. (**D**) Bar graph showing the coefficients of variation (CV) obtained for model output distributions of *t_MOMP_* when using 3×10^4^ Bcl-2/cell as the mean [Bcl-2]_0_ (striped bars; reproduced from Figure 4B), or when the average [Bcl-2]_0_ was changed to 6×10^4^ Bcl-2/cell (solid bars). As in Figure 4, proteins were classified as affecting the pre-MOMP rate of initiator caspase activity (Rate; gray), the MOMP threshold (Threshold; purple) or post-MOMP processes (Post-MOMP; green).

HeLa cells are predicted to be highly sensitive to even modest increases in Bcl-2 concentrations above endogenous levels (Figure 3). To test this prediction, we expressed variable levels of GFP-Bcl-2 in HeLa cells and then monitored *t_MOMP_* using a live-cell reporter (IMS-RP, [23]) in cells exposed to 50 ng/ml TRAIL plus low-dose cycloheximide. At wild-type levels of Bcl-2, all cells died within 5 hr, but as GFP-Bcl-2 levels increased above 4×10^5^ molecules/cell (∼13-fold above wild-type), a sudden transition was observed in *t_MOMP_* such that cell death was blocked indefinitely (Figure 7A). Between 2×10^5^ and 4×10^5^ Bcl-2 molecules/cell we observed a region of variable fate, with a subset of cells undergoing MOMP and others surviving (Figure 7A, gray shaded region). To determine how this variability arises, we ran a series of simulations using correlated initial conditions and sampled GFP-Bcl-2 levels over the experimentally observed range of 10^4^ to 1.2×10^6^ molecules per cell. Simulations were run for 12 hr, well past the time at which the last cells died in experiments; any simulated cells that had not undergone MOMP by 12 hr were assumed to have survived. As in real cells, we observed a region of variable fate at intermediate levels of Bcl-2 (Figure 7B, gray shaded region) although Bcl-2 levels bounding the region of variable fate were ∼3–4-fold lower in simulations than in experiments, a discrepancy we attribute either to error in the measurement of absolute protein abundance or to imprecise model calibration. When we used simulation to compare initial protein concentrations in cells that are predicted to survive vs. cells that are predicted to die (within the region of variable fate), we observed that [Bax]_0_ differed the most: cells that died had higher [Bax]_0_ and those that survived had lower [Bax]_0_ (Figure 7C, double asterisks). Thus, whereas multiple proteins play a role in controlling variability in death time under wild-type conditions (Figure 4B–C), under conditions of moderate Bcl-2 over-expression, [Bax]_0_ becomes the primary regulator of cell fate (Figure 7C).

### Control over variability of time-to-death in a multi-dimensional landscape

Given these findings, we asked whether simply doubling Bcl-2 levels would change which proteins were most influential in controlling variability in *t_MOMP_*. Strikingly, simply doubling the average initial Bcl-2 concentration from 3×10^4^ to 6×10^4^ proteins per cell was sufficient to alter the sensitivity of *t_MOMP_* to variation in the levels of other apoptosis regulators proteins. At 3×10^4^ Bcl-2/cell, no single protein had a dominant effect on variability in *t_MOMP_* (Figure 4B) whereas at 6×10^4^ Bcl-2/cell, Bax and Bcl-2 had nearly three times greater impact than any other protein, and varying either Bax or Bcl-2 yielded nearly as much variability in *t_MOMP_* as did varying all proteins simultaneously (sampling independently; Figure 7D). This demonstrates that protein over-expression (and presumably also protein depletion) changes the relative importance of other proteins in control of *t_MOMP_*. We also note that doubling [Bcl-2]_0_ significantly increased the variability of *t_MOMP_*: CV increased from 0.25 to 0.36 (black bars in Figures 4B and 7D, and Figure S12 in Text S1). Thus, the contribution made by variation in the level of particular proteins to variability in outcome is not necessarily a constant: contributions of individual proteins can vary dramatically over biologically plausible concentration ranges. Such contextual dependence of protein sensitivity also shows how protein over-expression can be misleading with respect to identifying factors that regulate a phenotype under endogenous conditions.

**Figure 8:**
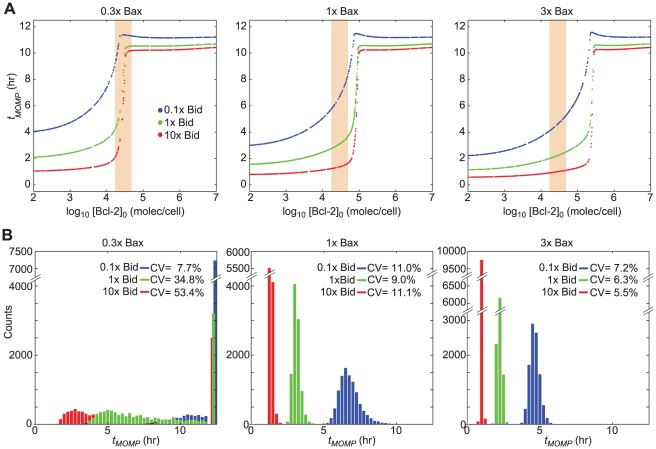
Variability in cell fate and time-to-death depends on the interplay between multiple factors. (**A**) Plots of *t_MOMP_* as a function of Bcl-2 level for three levels of Bax (0.3X [Bax]_0_, left, 1X [Bax]_0_, center, and 3X [Bax]_0_, right) and Bid (0.1X [Bid]_0_, blue, 1X [Bid]_0_, green, and 10X [Bid]_0_, red). Orange shading represents the 5^th^ and 95^th^ percentiles of the measured distribution of endogenous Bcl-2 in HeLa cells. (**B**) Histograms of the *t_MOMP_* distributions in the range of endogenous Bcl-2 (indicated by the orange shading) for varying Bid and Bax levels. For panels A and B, initial concentrations of Bcl-2 were sampled uniformly in the exponent between 10^2^ to 10^7^ molecules per cell and all other proteins concentrations were set at their default mean values (Table S2 in Text S3).

To further explore this context sensitivity, we focused on the joint control of *t_MOMP_* by three proteins that most influenced its variability in the model: Bcl-2, Bax, and Bid (Figure 4B). We changed [Bax]_0_ and [Bid]_0_ above and below default values in discrete three and ten-fold steps, respectively (the magnitude of these steps was chosen based on the sensitivity of *t_MOMP_* to the initial protein concentrations when evaluated under baseline conditions), while computing the relationship between *t_MOMP_* and [Bcl-2]_0_. We observed that changing [Bax]_0_ shifted the *t_MOMP_* vs. [Bcl-2]_0_ curves along the x-axis whereas increasing [Bid]_0_ shifted the curves along the y-axis and also changed the sharpness of the curves (Figure 8A). The net result was that changes in [Bax]_0_ affected the concentration of Bcl-2 at which cell fate switched from death to survival, whereas changes in [Bid]_0_ affected the mean and variance of *t_MOMP_*. The impact of natural variability in Bcl-2 expression (measured in HeLa cells; orange shading) on variance in *t_MOMP_* was greater at lower Bax levels (Figure 8B). For cells with normal [Bid]_0_, lowering [Bax]_0_ shifted the cells such that endogenous variability in [Bcl-2]_0_ created a huge spread in death time, with some cells surviving even at endogenous [Bcl-2]_0_ (Figure 8A–B, left). Taken together, these results demonstrate that the sensitivity of *t_MOMP_* to Bcl-2 levels is itself sensitive to the levels of two interacting proteins (a form of second-order sensitivity).

## Discussion

In this paper we examine the impact of naturally occurring variability in protein levels on variability in TRAIL-induced apoptosis. Our approach builds on previous work showing that variability in the timing and probability of death arises from cell-to-cell differences in protein levels that exist prior to TRAIL exposure and that this variability can therefore be modeled within a deterministic framework [15]. We make use of four features of apoptosis dynamics to explore the contributions made by variability in regulatory molecules to variability in the timing and efficiency of cell death. These dynamics were simulated by sampling from either independent or joint distributions whose variances were determined experimentally or estimated to represent the range of endogenous protein expression. Model parameters were not adjusted in this study to reproduce observed variability in responses to TRAIL; rather our ability to reproduce experimental data simply arose from substituting single values for initial conditions with log-normal distributions centered on previously determined EARM protein concentrations [15], [23], [29], [34]. Using sampling and feature-based sensitivity analyses, we find that multiple upstream proteins control the timing of death (*t_MOMP_* or *t_PARP_*) in HeLa cells, but that XIAP is the primary determinant of the rate and extent of death (*t_switch_* and *f_PARP_*, respectively). We also find that co-variation in protein levels reduces variability in death time, particularly when activator-inhibitor pairs are assumed to co-vary positively (e.g. Bcl-2 and Bax). Finally, we show through simulation and experiment that HeLa cells reside near a region of extreme sensitivity to Bcl-2 such that modest Bcl-2 over-expression causes cells to enter a region of parameter space associated with variable fate in which the primary determinants of phenotypic variability are quite different from those pertaining to normal conditions.

Correlations in the levels of different proteins across a population of single cells changes the apparent importance of specific proteins in controlling cellular phenotypes. Simulations based on measured correlations in protein concentrations appear to better represent the biology of real cells. However, we have found that such correlations can also have unexpected results because correlations can mask the biochemical roles of specific proteins. To date, we have only measured ten pairwise correlations (creating a joint distribution for five protein species) but in real cells, extrinsic noise will correlate all proteins to some degree unless they are actively regulated otherwise [35]. Using Equation 2 (Box 2), it is straightforward to estimate the potential impact of correlated protein expression (for pairs or larger sets of proteins) on model output and to prioritize measurement of those correlations with the greatest potential impact.

Under wild-type conditions, variability in the time at which a cell dies arises from variability in the concentrations of multiple regulatory molecules. Even perfect knowledge of the concentration of the model species that most strongly influences phenotype is only partially predictive of time-to-death because variability in other proteins makes a substantial contribution. In wild-type HeLa cells, knowledge of the eight most sensitive proteins is required to achieve a level of predictive ability (R^2^∼0.8) that can be achieved by experimentally measuring the rate of the single reaction corresponding to cleavage of the initiator caspase reporter protein [15]. This reflects the fact that a dynamic measurement reporting on a complex reaction has significantly more “information content” than a series of static measurements.

The ability of Bcl-2 to block apoptosis is well known [37], [38], [39] but our analysis sheds light on the precise mapping between the levels of Bcl-2 in individual cells and time of death. A relatively modest increase in Bcl-2 concentration (6-fold to 13-fold over endogenous Bcl-2 levels) causes cells to enter a region of parameter space associated with variable fate; in this region, Bax becomes the primary factor determining whether a cell lives or dies. A further increase in Bcl-2 over-expression (>13-fold) causes MOMP to be blocked indefinitely and corresponds approximately to the degree of Bcl-2 over-expression found in leukemic cells [40]. We note that in other cell types, this degree of over-expression might have no effect due to compensatory changes in the levels of other proteins in the apoptotic network. In Type I cells, for example, over-expression of Bcl-2 does not block death because MOMP is not needed to trigger apoptosis [41]. We have previously suggested that *t_PARP_* in HeLa cells is primarily determined by proteins controlling the rate of initiator caspase activation [15], but the results in this paper suggest that in other cells (e.g. those with slightly higher Bcl-2 levels than HeLa cells, Figure 7D), time-to-death may be primarily determined by other proteins, such as those that control the MOMP threshold. Whether the particular sensitivity of HeLa cells to natural variation in Bcl-2 and Bax levels confers a selective advantage or whether it is accidental cannot yet be determined.

The context dependence of classical and feature-based sensitivities is obvious mathematically but it is generally under-appreciated: sensitivity is not simply a function of network topology but also of position in parameter space. We show that “context dependence” is relevant over the natural range of protein concentrations found in populations of human cells. In HeLa cells, for example, variation in the levels of six proteins contributes roughly equally to variability in time of death under normal conditions, but when Bcl-2 levels are raised just two fold, only two proteins exert a significant impact. A clear implication is that we cannot consider experiments in which proteins levels are altered one at a time (by RNAi or over-expression) to represent univariate explorations of regulatory mechanism. Instead, protein over-expression and protein depletion shift cells in parameter space such that different proteins are dominant in controlling phenotype as compared to a wild-type context. This is true even if we consider only the immediate properties of the regulatory network and leave out the undoubtedly significant compensatory effects that occur at the level of other cellular pathways. Thus, the importance of specific proteins in a regulatory pathway is likely to be mis-estimated based on univariate and qualitative assessment of experimental perturbation; quantitative, system-level approaches promise to be more accurate in this regard.

Non-genetic heterogeneity has recently emerged as an important topic in a variety of fields and it has become increasingly clear that cell-to-cell variability in protein expression is a key factor in a wide range of cellular decisions [1], [2], [3], [4], [5], [6], [7], [8], [9], [10], [11], [12], [13], [14], [15]. We expect the approach described here for analyzing cell-to-cell variability in receptor-mediated apoptosis to be generally useful in the analysis of other signaling systems in which phenotypic variability is observed. This is particularly true in those cases in which pre-existing variation in protein levels is a dominating influence and it is appropriate to use deterministic modeling coupled to Monte-Carlo procedures for sampling parameter distributions.

## Methods

### Cell culture and transfections

HeLa cells were maintained in DMEM (Mediatech, Inc) supplemented with L-glutamine (Gibco), penicillin/streptomycin (Gibco) and 10% fetal bovine serum (FBS, Mediatech, Inc). FuGENE 6 (Roche) was used to transfect HeLa cells expressing IMS-RP [23] with a pExchange vector (Stratagene) into which we cloned a cDNA for EGFP-Bcl-2. Stable EGFP-Bcl-2 transfectants were isolated by selecting with neomycin and sorted on a FACSAria (BD Biosciences) to sample expression levels across a wide range.

### Live-cell microscopy

HeLa cells expressing IMS-RP and GFP-Bcl-2 were plated in a 96-well glass bottom plate (Matrical). For all live-cell microscopy experiments, cells were treated with 50 ng/ml Superkiller TRAIL (Alexis Biochemicals) and 2.5 ug/ml cycloheximide (Sigma-Aldrich) and imaged on a Nikon TE2000E at 20× magnification with frames every 5 min in a 37°C humidified chamber in phenol-red free CO_2_-independent medium (Invitrogen) supplemented with 1% FBS, L-Glutamine, and Penicillin/Streptomycin). GFP-Bcl-2 fluorescence was quantified at *t* = 0 (time of treatment) by manually outlining the cell and measuring the average fluorescence intensity within the outline. MOMP was scored manually by monitoring cytosolic translocation of IMS-RP. To convert the *x*-axes in Figure 6A to proteins/cell, the average GFP fluorescence intensity at *t* = 0 was set equal to the average number of GFP-tagged proteins per cell as measured by quantitative immunoblotting (Figure S5 in Text S1).

### Flow cytometry

Distributions of initial protein levels were measured in untreated HeLa cells (fixed with 4% paraformaldehyde and permeabilized with methanol) on a FACSCalibur (BD Biosciences). Antibodies were carefully validated as described in Figures S6, S7 in Text S1 and the following antibodies were found to be suitable for measurement of total protein levels: α-Bid (HPA000722, Atlas Antibodies), α-Bax (MAB4601, Chemicon International), α-Bcl-2 (SC7382 and SC783, Santa Cruz Biotechnology), α-XIAP (610717, BD Biosciences), α-caspase-3 (SC7272, Santa Cruz Biotechnology). Correlations in protein levels were measured by combining pairs of antibodies generated in different species or pairing fluorophore-conjugated version of the primary antibodies listed above (α-caspase-3-AF488, α-Bax-PE, α-XIAP-AF647). Cells were gated in both forward scatter and side scatter to select a population of cells of similar size and the data analyzed in MatLab (Mathworks).

### Modeling

Simulations were run in Jacobian (RESgroup) using the EARM1.3 ordinary differential equation model. EARM1.3 is an extension of the original EARM1.1 [29], modified to include general protein synthesis and degradation as described previously [15]. This model had been manually calibrated to represent the response of a single HeLa cell to TRAIL treatment [18]. Lists of reactions, initial protein concentration and parameter values are included in Tables S1, S2, S3, S4 in Text S3.

When sets of simulations called for sampling from distributions of initial protein levels, we used a custom Perl script to generate series of random numbers that were sampled from a multivariate normal distribution with specified variances and co-variances (see Tables S2 and S3 in Text S3). These number series were then transformed to achieve the final log-normally distributed series with appropriate means and coefficients of variation.

To calculate feature sensitivities numerically, series of simulation pairs were run for each protein by sampling its initial concentration uniformly in the exponent for values between 10^2^ to 10^7^ proteins per cell and then running simulations using 100% and 101% of this value, setting all other initial protein concentrations and rate constants at their default value. Sensitivities were then calculated using:
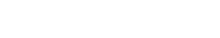
where *k_100_* and *k_101_* are the values of the sampled initial protein concentration for the simulation pair (100% and 101%, respectively) and similarly *q_100_* and *q_101_* are the values of feature *q* using 100% and 101% of the sampled initial protein concentration, respectively.

## Supporting Information

Text S1 **Supporting experimental and computational results.** This text contains Figures S1, S2, S3, S4, S5, S6, S7, S8, S9, S10, S11, S12, a compilation of additional experimental and computational results.
(PDF)

Text S2 **Derivation of equations.** This text contains explanatory notes on the relationships between feature-based and conventional sensitivities and on how sensitivities propagate variance in initial protein concentration to variance in pathway outputs as well as the derivations of Box 1 Equation 1 and Box 2 Equation 2.
(PDF)

Text S3 **Description of EARM1.3.** This text contains Tables S1, S2, S3, S4 which list model reactions (Table S1), initial protein concentrations (averages and coefficients of variation; Table S2), protein covariances (Table S3) and parameter values (Table S4) used in EARM1.3.
(PDF)
