# Supplementary material for: Exploring the Contextual Sensitivity of Factors that Determine Cell-to-Cell Variability in Receptor-Mediated Apoptosis
Source: PLoS Comput Biol. 2012 Apr 26;8(4):e1002482. doi: 10.1371/journal.pcbi.1002482 (PMC3343095; doi:10.1371/journal.pcbi.1002482)
Supplement: Text S1 — Supporting experimental and computational results. This text contains Figures S1, S2, S3, S4, S5, S6, S7, S8, S9, S10, S11, S12, a compilation of additional experimental and computational results. (PDF) [file pcbi.1002482.s001.pdf]

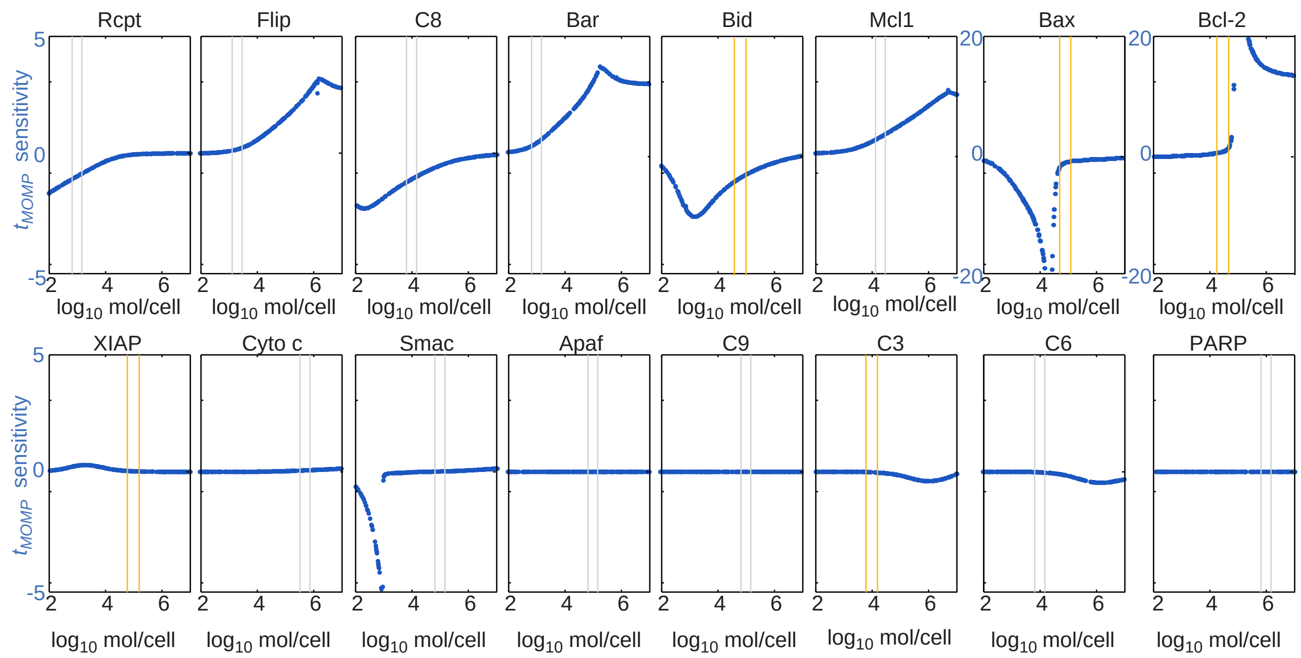


**Figure S1. Analytically calculated sensitivity of $t_{MOMP}$ to changes in protein initial concentrations.** Scatter plots show the simulated relationship between initial protein concentration and $t_{MOMP}$ sensitivity, as calculated using the analytical form described in Equation 1 (see also Figure Box 1 for the specific expression for $t_{MOMP}$. The initial concentration for the indicated protein was uniformly sampled in the exponent for values between $10^2$ to $10^7$ proteins per cell while all other initial protein concentrations and rate constants were set at their default value. Vertical bars represent the 5th and 95th percentiles of the measured (orange, see Figure 3 and Figure S8 in this Text S1) or assumed (gray, see Table S2 in Text S3 for mean and CV) distributions in endogenous protein concentrations for untreated HeLa cells.

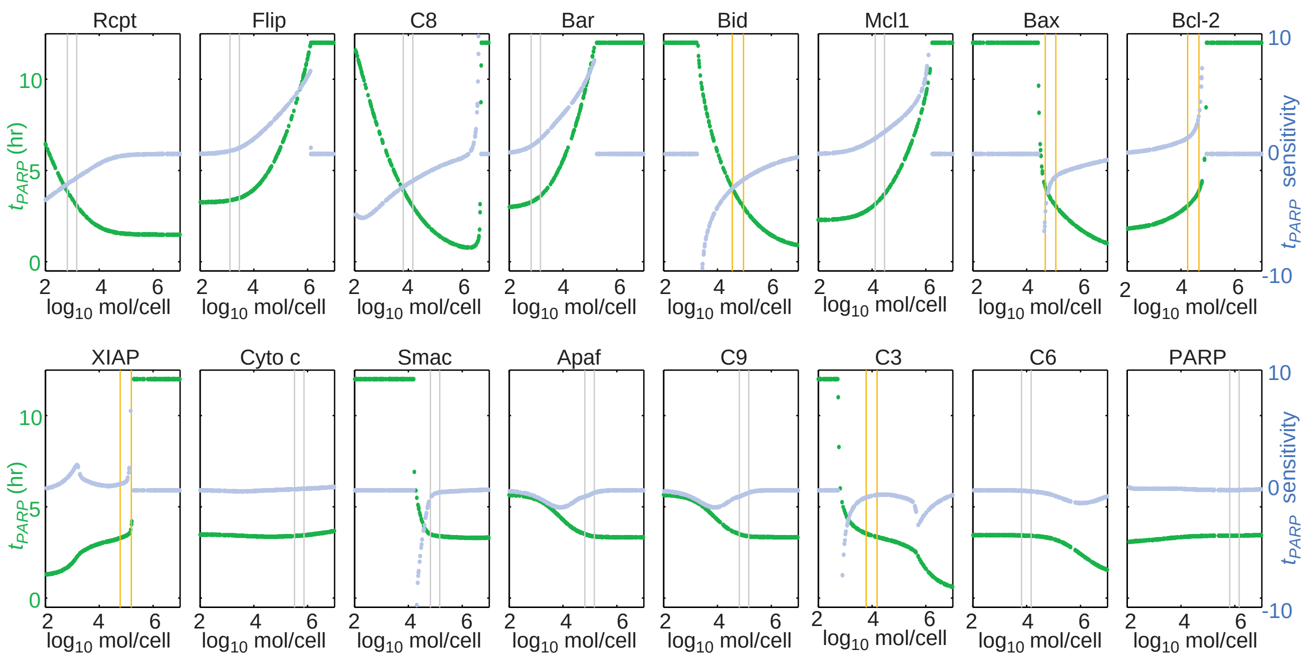


**Figure S2. Sensitivity of $t_{PARP}$ to changes in protein initial concentrations.** Scatter plots show the simulated relationship between initial protein concentration and $t_{PARP}$ (green) or numerically calculated $t_{PARP}$ sensitivity (blue) following TRAIL addition, for the indicated proteins. The initial concentration for the indicated protein was uniformly sampled in the exponent for values between $10^2$ to $10^7$ proteins per cell while all other initial protein concentrations and rate constants were set at their default value. Vertical bars represent the 5th and 95th percentiles of the measured (orange, see Figure 3 and Figure S8 in this Text S1) or assumed (gray, see Table S2 in Text S3 for mean and CV) distributions in endogenous protein concentrations for untreated HeLa cells.

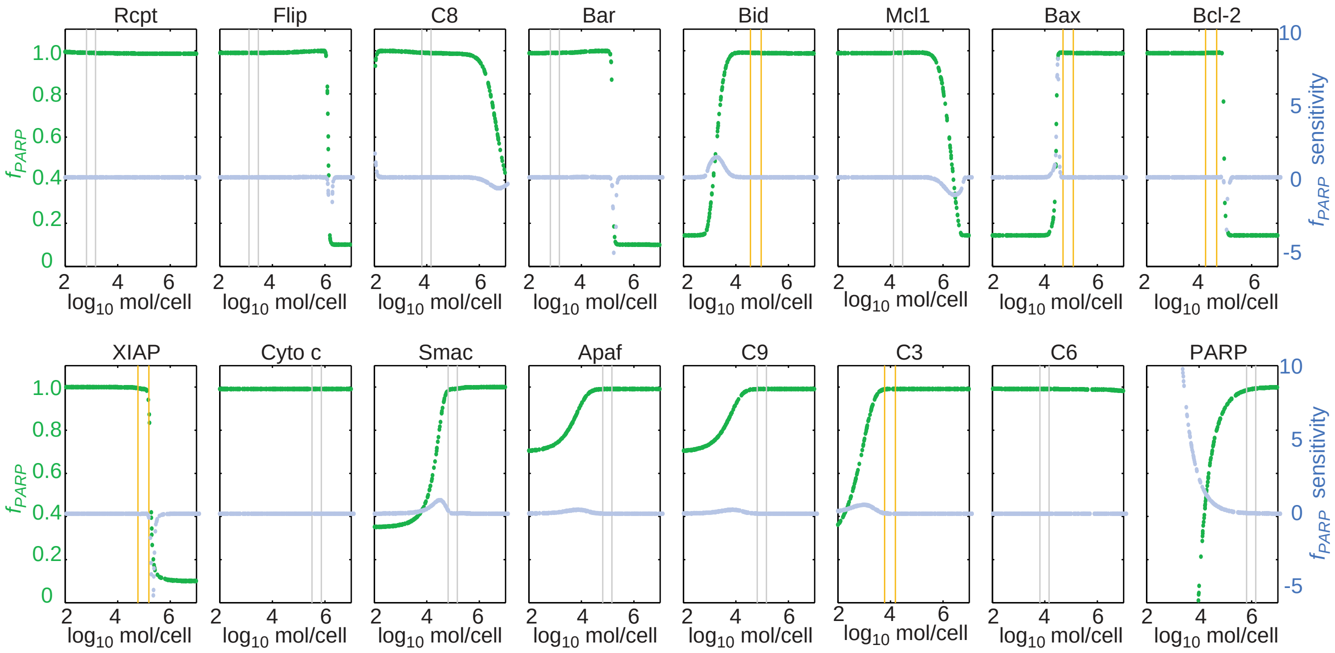


**Figure S3. Sensitivity of $f_{PARP}$ to changes in protein initial concentrations.** Scatter plots show the simulated relationship between initial protein concentration and $f_{PARP}$ (green) or numerically calculated $f_{PARP}$ sensitivity (blue) following TRAIL addition, for the indicated proteins. The initial concentration for the indicated protein was uniformly sampled in the exponent for values between $10^2$ to $10^7$ proteins per cell while all other initial protein concentrations and rate constants were set at their default value. Vertical bars represent the 5th and 95th percentiles of the measured (orange, see Figure 3 and Figure S8 in this Text S1) or assumed (gray, see Table S2 in Text S3 for mean and CV) distributions in endogenous protein concentrations for untreated HeLa cells.

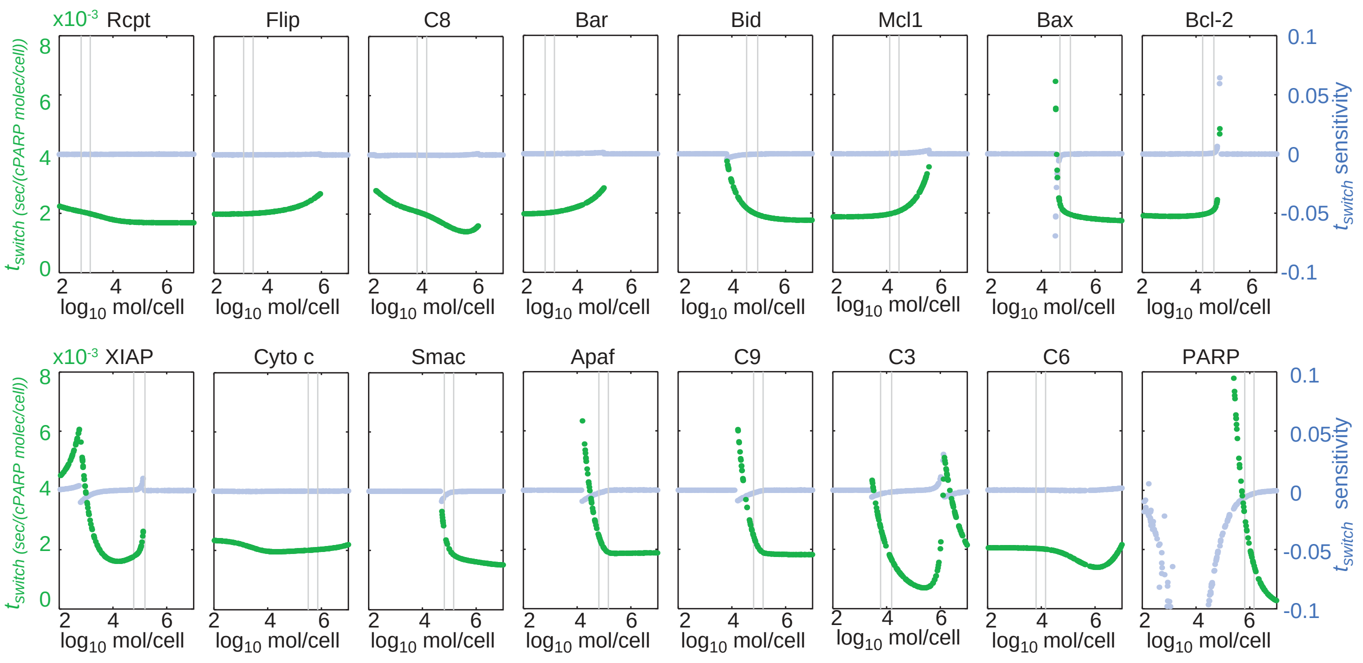


**Figure S4. Sensitivity of $t_{switch}$ to changes in protein initial concentrations.** Scatter plots show the simulated relationship between initial protein concentration and $t_{switch}$ (green) or numerically calculated $t_{switch}$ sensitivity (blue) following TRAIL addition, for the indicated proteins. The initial concentration for the indicated protein was uniformly sampled in the exponent for values between $10^2$ to $10^7$ proteins per cell while all other initial protein concentrations and rate constants were set at their default value. Vertical bars represent the 5th and 95th percentiles of the measured (orange, see Figure 3 and Figure S8 in this Text S1) or assumed (gray, see Table S2 in Text S3 for mean and CV) distributions in endogenous protein concentrations for untreated HeLa cells. Note that when a simulated cell does not die (see $f_{PARP}$ response curves, Figure S3 in this Text S1), $t_{switch}$ is assigned an arbitrarily high value.

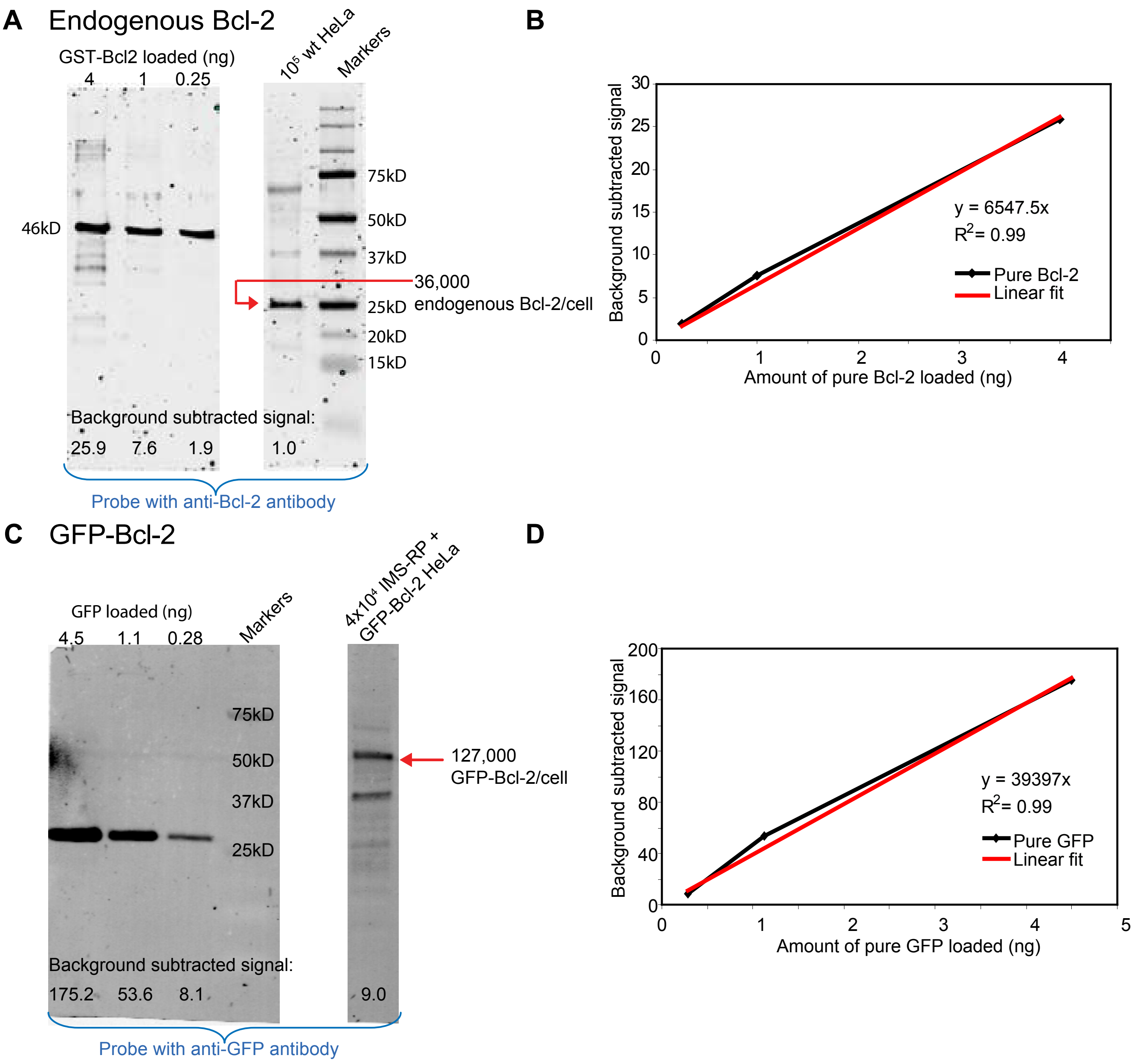


**Figure S5. Immunoblot quantification of endogenous Bcl-2 and GFP-Bcl-2 in HeLa cells.** Pure protein and HeLa cell lysate were loaded on a 10% Tricine SDS-PAGE gel. After transfer to a PVDF membrane, blots were probed, scanned on a LI-COR Odyssey, and quantified digitally. **A**) The membrane was probed with rabbit anti-Bcl-2 (Santa Cruz Biotechnology SC783) followed by AF680-conjugated anti-rabbit. Pure Bcl-2 is a 46 kDa fusion protein (Santa Cruz Biotechnology SC4096). **B**) From the standard curve, we calculate that, on average, a single HeLa cell has $1.57 \times 10^{-15}$ g Bcl-2. Using a molecular weight of 26,135 Da for Bcl-2, we find 36,000 Bcl-2/cell. The average of 14 such measurements yields 30,000 Bcl-2/HeLa cell; s.e.m = 10,000. **C**) The membrane was probed with mouse anti-GFP (Roche #11814460001) followed by IRDye 800-conjugated anti-mouse. Pure GFP was purchased from Biovision (#4999-100). **D**) From the standard curve, we calculate that, on average, an IMS-RP GFP-Bcl-2 HeLa cell has $5.68 \times 10^{-15}$ g GFP-Bcl-2. Using a molecular weight of 27,000 Da for GFP, we find 127,000 GFP-Bcl-2/cell. The average of 5 such measurements yields 133,000 GFP-Bcl-2/HeLa cell, s.e.m = 18,000. We set the background-subtracted average GFP-Bcl-2 fluorescence intensity of the population of cells in the first frame of the movie used in Figure 7 equal to the average number of GFP-Bcl-2 in the HeLa cells (calculated above). By adding 30,000 endogenous Bcl-2 to this amount, the x-axis of Figure 7A was rescaled into total Bcl-2 proteins per cell.

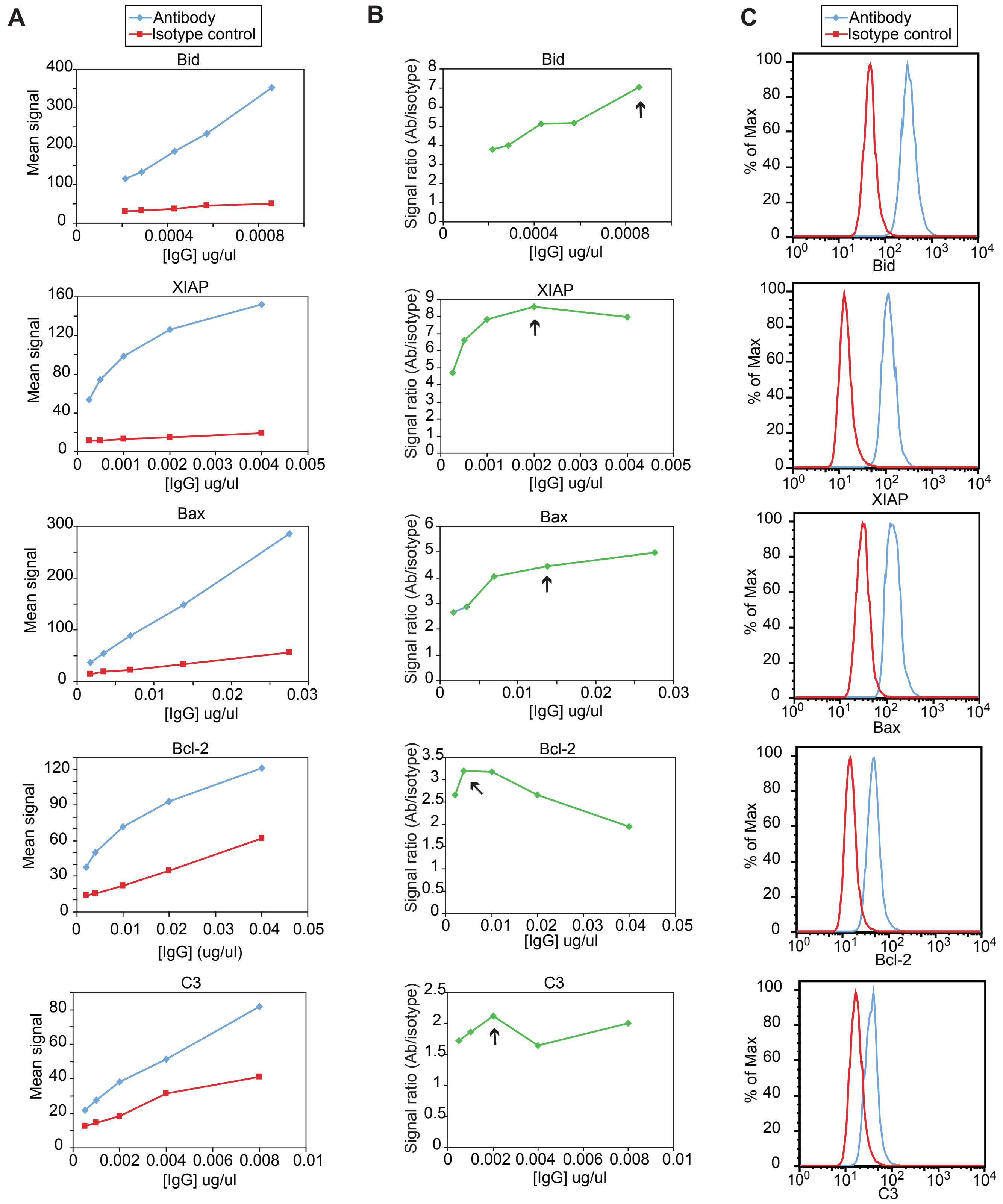


**Figure S6. Optimization and validation of antibody dilutions for flow cytometry.** **A**) Plots showing average flow cytometry signal as a function of increasing concentrations of antibody and isotype control. For Bid, the matched isotype control was rabbit IgG; for XIAP, Bax, and Bcl2, mouse $IgG_1$; for C3, mouse $IgG_{2a}$ (Santa Cruz Biotechnology). **B**) Plots showing the ratio in average signal from antibody staining versus background isotype control. Arrows indicate the concentrations of antibodies used in all other experiments. **C**) Histograms showing the degree of overlap of distributions of isotype control and antibody-stained cells. All the antibodies used are described in the Methods section of the main text.

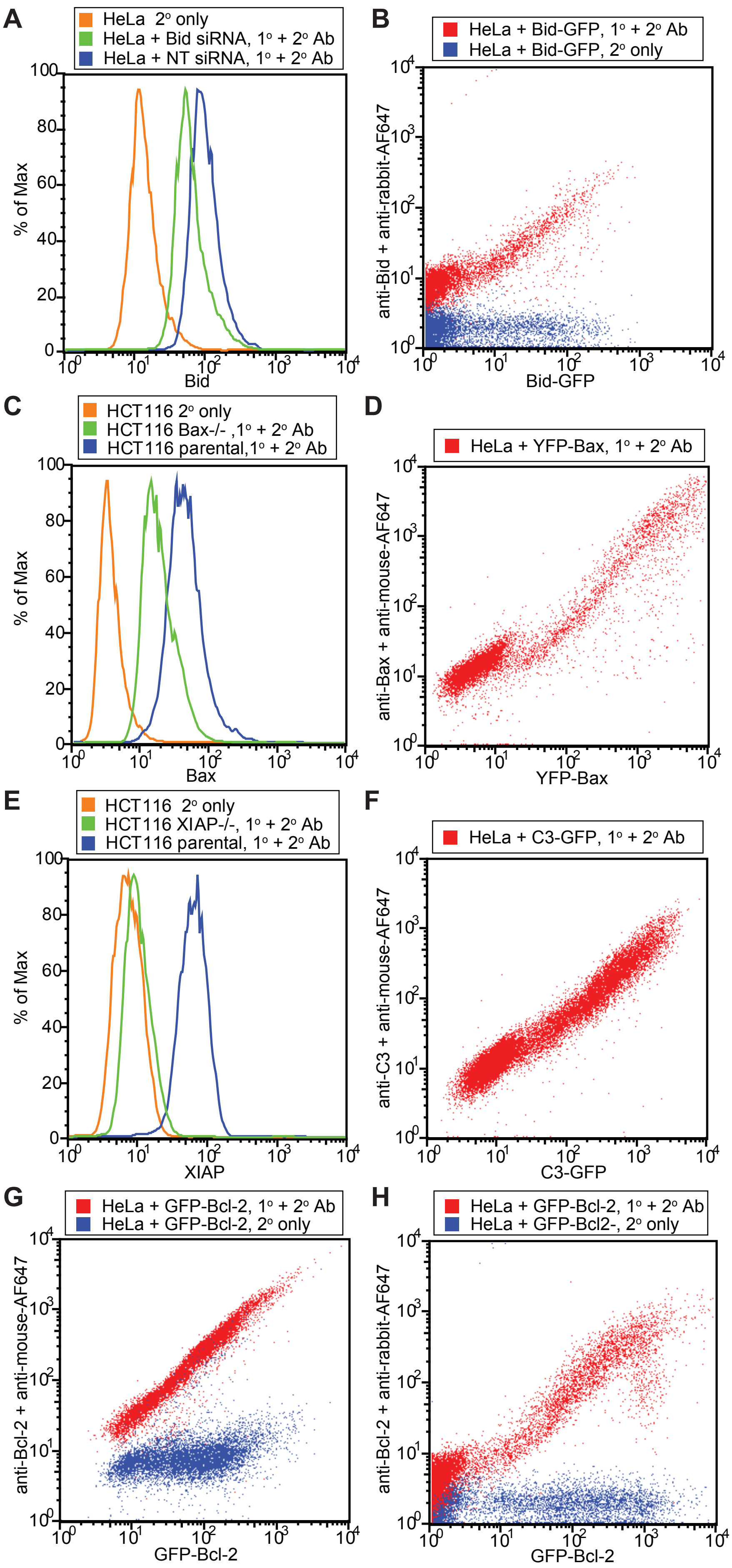


**Figure S7. Validation of antibodies used for measuring protein distributions by flow cytometry.**

**A-B**) Validation of a rabbit anti-Bid antibody (Atlas Antibodies HPA000722). **A**) Histograms of anti-Bid stained non-targeting (NT) siRNA treated HeLa cells (blue) compared to anti-Bid stained, Bid siRNA treated cells (green) and cells stained with secondary antibody only (orange). **B**) 2D scatter plot of anti-Bid vs. Bid-GFP signal in Bid-GFP transfected HeLa cells stained with anti-Bid followed by Alexa Fluor 647 (AF647) conjugated anti-rabbit secondary antibody (red), or secondary antibody only (blue).

**C-D**). Validation of mouse anti-Bax antibody (Chemicon International MAB4601). **C**) Histograms of parental HCT116 cells stained with anti-Bax followed by a secondary antibody (blue) or with secondary antibody alone (orange) and of HCT116 Bax-/- derivatives, stained with both anti-Bax and secondary antibody (green). **D**) 2D scatter plot of anti-Bax vs. YFP-Bax signal in YFP-Bax transfected HeLa stained with anti-Bax and AF647-conjugated anti-mouse antibody.

**E**) Validation of mouse anti-XIAP antibody (BD Biosciences 610717); histograms of parental HCT116 cells stained with anti-XIAP and secondary antibody (blue) or with secondary antibody alone (orange) and HCT116 XIAP-/- cells stained with anti-XIAP and secondary antibody.

**F**) Validation of mouse anti-C3 antibody (Santa Cruz Biotechnology SC7272); 2D scatter plot of anti-C3 vs. C3-GFP signal in C3-GFP transfected HeLa cells stained with anti-C3 and AF647 conjugated secondary antibody.

**G-H**) Validation of mouse anti-Bcl-2 antibody (Santa Cruz Biotechnology SC7382; (G)) and rabbit anti-Bcl-2 antibody (Santa Cruz Biotechnology SC783; (H)) in 2D scatter plots of anti-Bcl-2 vs. GFP-Bcl-2 signal for GFP-Bcl-2 transfected HeLa cells stained with anti-Bcl-2 and AF647 conjugated secondary antibody.

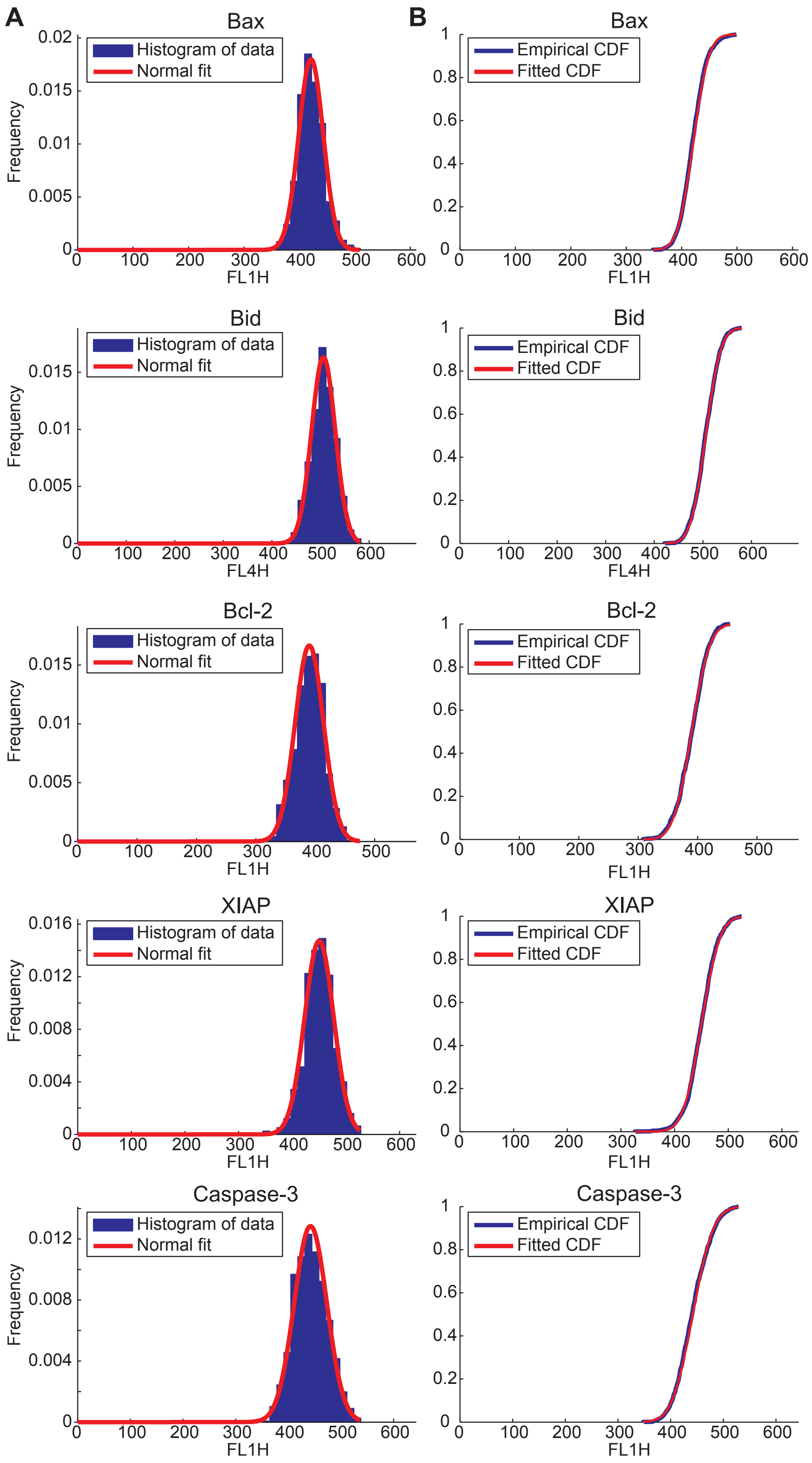


**Figure S8. Protein level distributions are well fit by lognormal distributions.** Fitted probability density functions and measured histograms (**A**), and empirical and fitted cumulative density functions (**B**) of fluorescence intensity in HeLa cells stained with validated antibodies against the indicated proteins, as measured by flow cytometry. The data, recorded in a .fcs file, were read into MATLAB in linear form and gated to select cells of similar size as described in Methods. All distributions were found to pass the Kolmogorov-Smirnov test for normality indicating that the underlying fluorescence distributions are well fit by lognormal distributions.

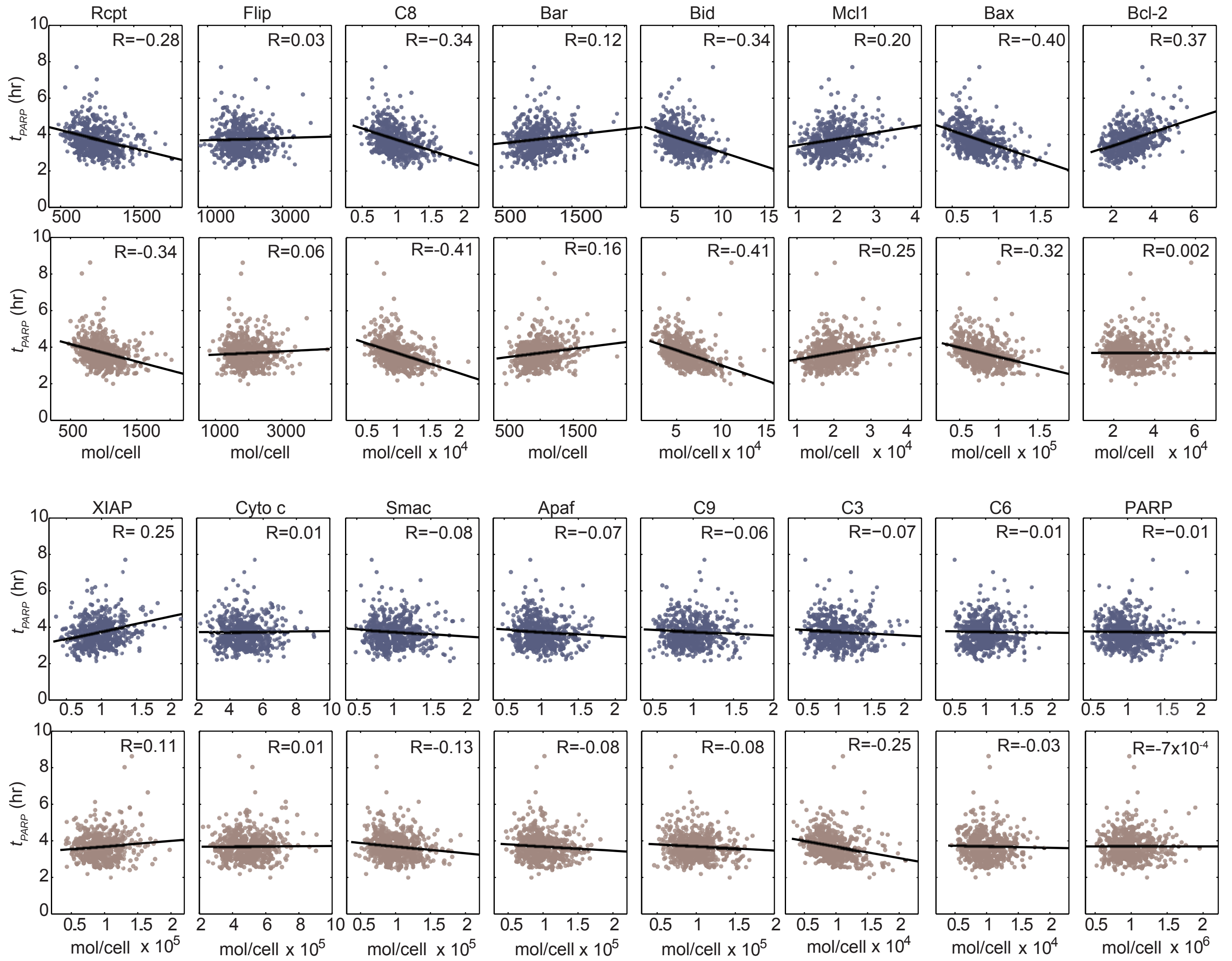


**Figure S9. Correlations in initial conditions qualitatively and quantitatively impact the relationship of time of death to protein concentration.** Scatter plots of time of death ($t_{PARP}$) vs. initial concentration for the indicated proteins for 200 simulations of the model with independently sampled initial conditions (blue) or initial conditions correlated as measured for Bax, Bcl2, Bid, caspase-3 and XIAP and all other proteins varying independently (brown). Black lines are linear regressions of the data points, and R values are the Pearson correlation coefficients.

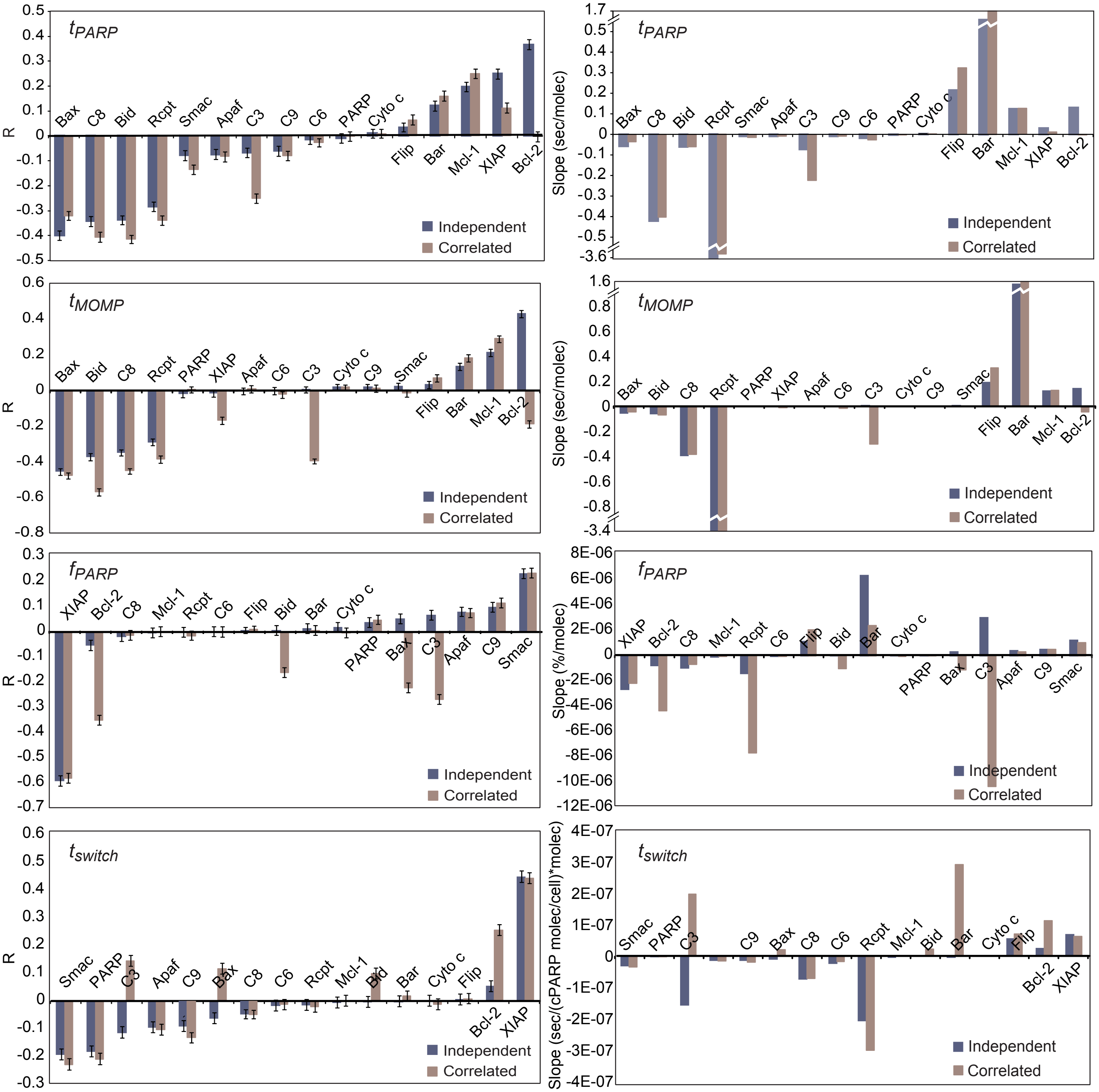


**Figure S10. Correlation in protein levels affects the rank ordering of impact of species on all four features**. Bar graphs of the Pearson correlation coefficients (R; left) and slopes of the linear regression (right) of each feature against endogenous variability in each protein listed as obtained for simulations sampling from fully independent distributions (blue), from joint distribution for Bax, Bcl-2, Bid, caspase-3 and XIAP and all other proteins sampled independently (brown). For each feature, the protein species were ordered by the value of R. The bar graph showing the Pearson correlation coefficients for $t_{PARP}$ (top left) is reproduced from Figure 5E to allow comparisons of the effects across different features.

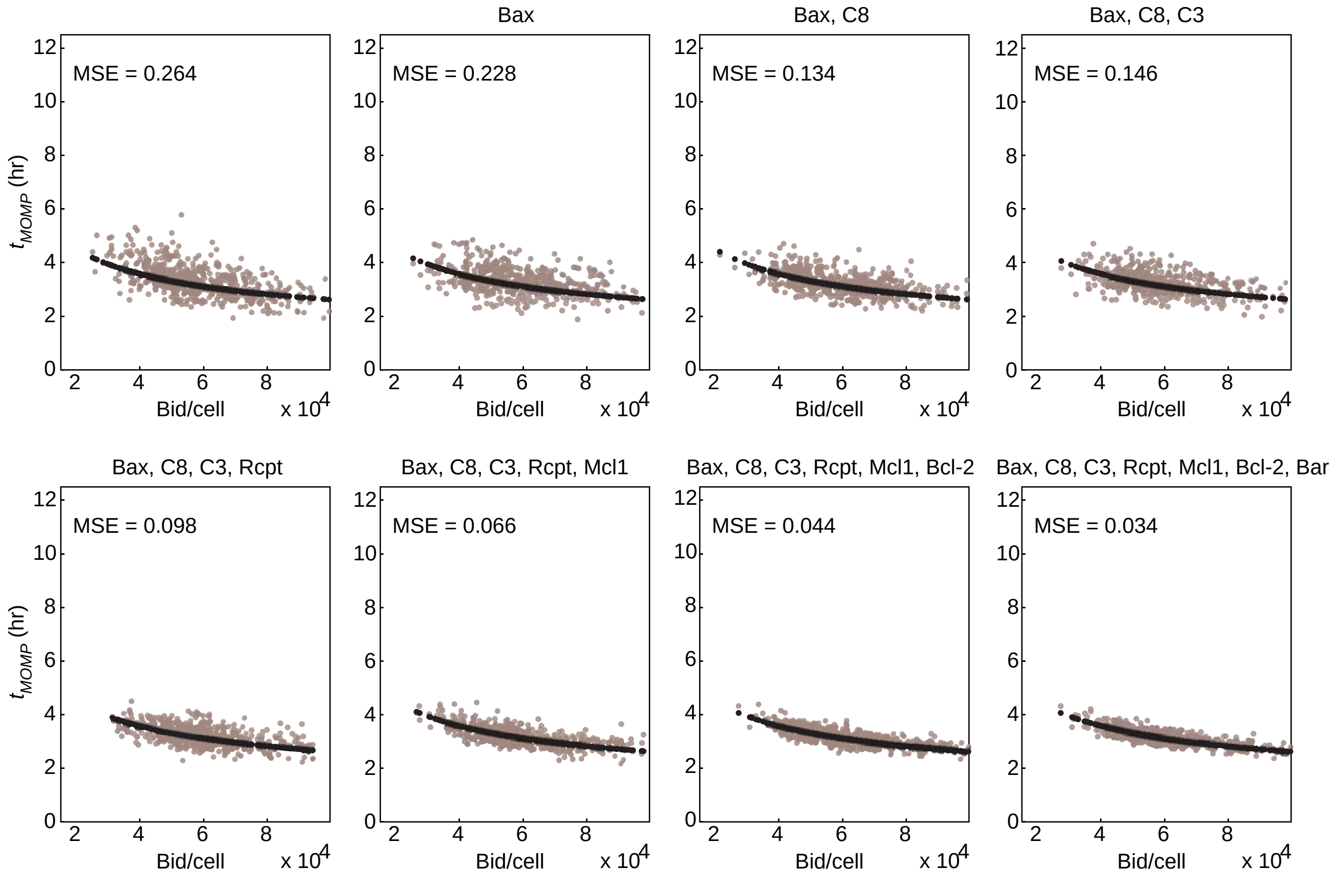


**Figure S11. Predictability of death time is improved by knowledge of key protein concentrations.** Scatter plots of predicted $t_{MOMP}$ as a function of Bid initial protein levels either with perfect knowledge of the concentrations of all model species (black points) or knowing only the levels of the most influential proteins listed above the plot (ranked by $R^2$ for $t_{MOMP}$ as in Figure S10 in this Text S1; brown points). Simulations were selected from a series for which initial protein concentrations were sampled from joint distribution for Bax, Bcl-2, Bid, caspase-3 and XIAP (all other proteins sampled independently) by defining "knowledge" of a protein as having an initial concentration within the range of average ± 12.5%. MSE is the mean squared error relative to perfect knowledge (black points). These graphs show example results from a single run while the curves in Figure 6B show average results and standard deviations for n = 10 runs.

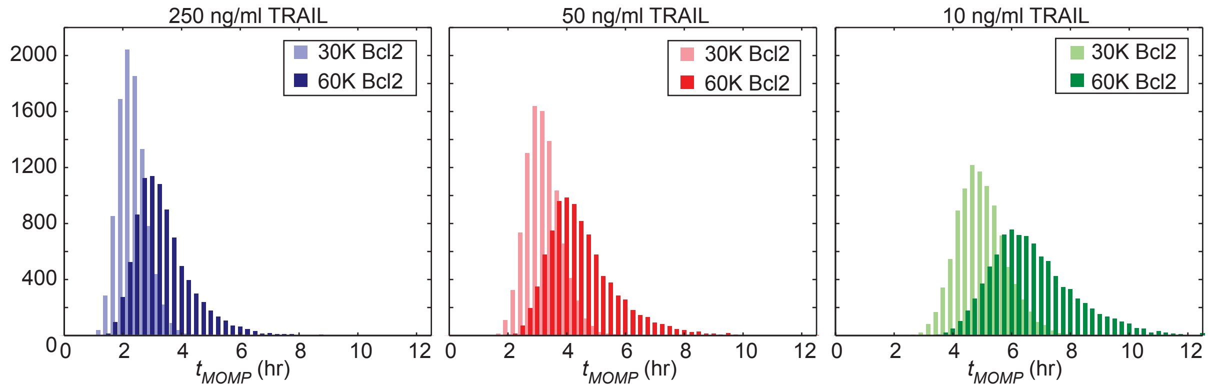


**Figure S12. Sensitivity of $t_{MOMP}$ variability to doubling the initial level of Bcl-2.** Histograms of the distributions of $t_{MOMP}$ as observed in simulation with average Bcl-2 levels set to 30,000 per cell (light bars) or 60,000 per cell (dark bars), for three doses of TRAIL (2.5ug/ml cycloheximide). Bax, Bcl2, Bid, Caspase-3 and XIAP initial protein initial concentrations were sampled from the measured joint distribution and all other initial concentrations were independently from lognormal distributions parametrized as described in Table S2 in Text S3. Distributions generated using 30,000 Bcl-2 per cell better recapitulate those observed experimentally (see Spencer et al., (2009), Ref. 15 in main text for experimentally observed $t_{MOMP}$ distributions).
