# Supplementary material for: Exploring the Contextual Sensitivity of Factors that Determine Cell-to-Cell Variability in Receptor-Mediated Apoptosis
Source: PLoS Comput Biol. 2012 Apr 26;8(4):e1002482. doi: 10.1371/journal.pcbi.1002482 (PMC3343095; doi:10.1371/journal.pcbi.1002482)
Supplement: Text S2 — Derivation of equations. This text contains explanatory notes on the relationships between feature-based and conventional sensitivities and on how sensitivities propagate variance in initial protein concentration to variance in pathway outputs as well as the derivations of Box 1 Equation 1 and Box 2 Equation 2. (PDF) [file pcbi.1002482.s002.pdf]

TEXT S2 – DERIVATION OF EQUATIONS

The Relationship Between Featured-Based and Conventional Sensitivities

A system of ordinary differential equations (ODE) involving one dynamical variable and its conventional sensitivity are defined in the following way:

$$\dot{y} = y(t;k)$$

$$\dot{S}_{y,k} = \frac{\partial y(t;k)}{\partial k}$$

where y is a dynamical variable, the dot notation of $\dot{y}$ signifies a time derivative, and $\dot{S}_{y,k}$ is the sensitivity of y with respect to the parameter k. Solving these equations yields the trajectories over time of the dynamical variable and of its sensitivity, respectively,.

Here we consider two time-based features t_{delay} and t_{switch}, as well as their respective sensitivities. In the context of extrinsice cell death for example, we can let y represent cPARP, then the definition of t_{delay} is given by the time at which cPARP has reached a predefined threshold $y_{threshold}$.

$$y(t_{delay}) \equiv y_{threshold} \quad \text{(Eq. S1)}$$

Note that t_{delay} is defined implicitly. The switching time t_{switch} is given by the inverse of the slope of the trajectory at t_{delay}.

$$t_{switch} \equiv \left(\left. \frac{\partial y}{\partial t} \right|_{t_{delay}} \right)^{-1} \text{(Eq. S2)}$$

If the dynamical variable y represents number of cPARP (as we use in EARM), then the units of the right hand side are of time. The sensitivities of these two time-based features are given by the following two expressions:

$$\dot{S}_{t_{delay};k} \equiv \frac{\partial t_{delay}}{\partial k}$$

$$\dot{S}_{t_{switch};k} \equiv \frac{\partial t_{switch}}{\partial k}$$

In order to obtain the sensitivity of t_{delay}, we take the full derivative of Equation S1 with respect to the parameter k and evaluate the expression at $t = t_{delay}$, noting that t_{delay} has an implicit dependence on k and that the left hand side is constant (from the definition of t_{delay} in Eq. S1) so must evaluate to 0.

$$\left. \frac{dy}{dk} \right|_{t=t_{delay}} = \left. \frac{\partial y}{\partial k} \right|_{t=t_{delay}} + \left. \frac{\partial y}{\partial t} \right|_{t=t_{delay}} * \frac{\partial t_{delay}}{\partial k}$$

$$= 0$$

Nevertheless, this expression can be rearranged to yield the sensitivity of t_{delay}:

$$\frac{\partial t_{delay}}{\partial k} = -\left.\frac{\partial y}{\partial k}\right|_{t=\, t_{delay}} \left(\left.\frac{\partial y}{\partial t}\right|_{t=\, t_{delay}}\right)^{-1} \quad \text{Eq. S3}$$

Equation S3 has an appealing geometric interpretation. The three fractions of Equation S3 are related to the three legs of a triangle in the y vs. t plot (Figure) in the following way: the slope of the hypotenuse is given by the slope of the trajectory at $t = t_{delay}$, which is simply the time derivative of the dynamical variable evaluated at $t = t_{delay}$. But the slope itself is the ratio of the length of the vertical leg to the length of the horizontal leg, which are given respectively by the conventional sensitivity of y with respect to k and the feature-based sensitivity.

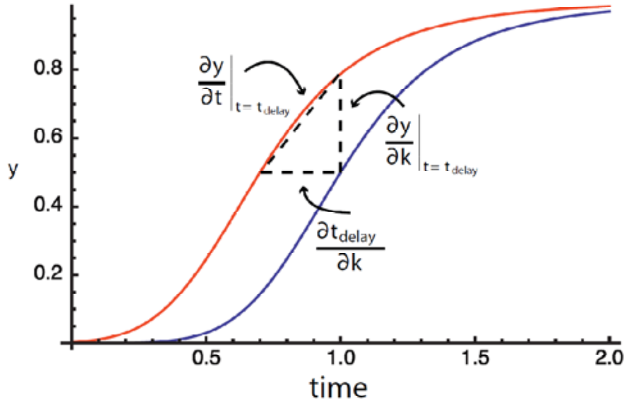

Figure. Geometrical interpretation of the sensitivity of t_{delay}.

To obtain the sensitivity of t_{switch}, we simply take the partial derivative with respect to k as there is no implicit dependence on t_{switch}.

$$\frac{\partial t_{switch}}{\partial k} = -\left(\left.\frac{\partial y}{\partial t}\right|_{t=\, t_{delay}}\right)^{-2} \left.\frac{\partial S_{y;k}}{\partial t}\right|_{t=\, t_{delay}}$$

Sensitivities Propagate Variance in Initial Protein Concentrations to Variance in Pathway Output

A population of heterogenous cells with varying protein content can be modeled as a collection of systems of ODEs, each instantiation with unique initial protein concentrations representing an individual cell. The following system of ODEs represents a single cell.

$$\dot{y}_i = g\left(t, \{y_j\}, \{k_l\}, \{y_{0,i}\}\right)$$

$$y_i(0) = c_{0,i}$$

The dynamical variables y_i represent the levels of proteins, protein complexes and species with post-translational modifications. Initial protein concentrations vary from cell to cell but have been measured and are well-described by a multivariate log normal distribution.

$$p(\{y_{0,i}\}) \prod_{i=1}^{N} d\left(\log c_{0,i}\right) = \sqrt{\frac{1}{Det\sigma_{k,l}^{0}}}\, exp\left[-\frac{1}{2}\left(\log c_{0,k} - \langle \log c_{0,k}\rangle\right)\left(\sigma_{k,l}^{0}\right)^{-1}\left(\log c_{0,l} - \langle \log c_{0,l}\rangle\right)\right] \prod_{i=1}^{N} d\left(\log c_{0,i}\right) \quad \text{Eq. S4}$$

The measured mean log levels of the ith protein is given by $\langle \log c_{0,i}\rangle$, and the variable $\sigma_{k,l}^{0}$ denotes the measured covariances of the log concentrations. The population behavior for variable y_i is easy to write down formally.

$$p(\{y_i(t)\}) \equiv \langle \delta\left(y_i(t) - \hat{y}_i\left(t; \{c_{0,i}\}\right)\right)\rangle = \int_0^\infty \prod_{i=1}^{N} d\left(\log y_{0,i}\right) p\left(\{c_{0,i}\}\right) \delta\left(y_i(t) - \hat{y}_i\left(t; \{c_{0,i}\}\right)\right) \quad \text{Eq. S5}$$

But because the ODEs are nonlinear, a closed form for Equation S5 is generally impossible and one must settle for moment expansions.

Taylor expanding y_i and performing an average with Equation S4 yields the following:

$$\langle y_i(t)\rangle \approx y_i\left(t; \{\langle \log c_{0,j}\rangle\}\right) + \frac{\partial y_i}{\partial\left(\log c_{0,j}\right)}\langle \log c_{0,j}\rangle + \frac{1}{2}\frac{\partial^2 y_i}{\partial \log c_{0,j}\, \partial \log c_{0,k}}\langle \log c_{0,j} \log c_{0,k}\rangle$$

The second moment is given by

$$\langle y_i(t) y_j(t)\rangle - \langle y_i(t)\rangle\langle y_j(t)\rangle$$

Evaluating its Taylor expansion to second order yields the expression

$$\frac{\partial y_i}{\partial\left(\log c_{0,k}\right)} \frac{\partial y_j}{\partial\left(\log c_{0,l}\right)} \langle \log c_{0,k} \log c_{0,l}\rangle - \langle \log c_{0,k} \log c_{0,l}\rangle = \frac{\partial y_i}{\partial\left(\log c_{0,k}\right)}\, \sigma_{k,l}^{0}\, \frac{\partial y_j}{\partial\left(\log c_{0,l}\right)}$$

The last expression is the covariance (or variance, if $i = j$) of the model outputs.
