# Supplementary material for: Exploring the Contextual Sensitivity of Factors that Determine Cell-to-Cell Variability in Receptor-Mediated Apoptosis
Source: PLoS Comput Biol. 2012 Apr 26;8(4):e1002482. doi: 10.1371/journal.pcbi.1002482 (PMC3343095; doi:10.1371/journal.pcbi.1002482)
Supplement: Text S3 — Description of EARM1.3. This text contains Tables S1, S2, S3, S4 which list model reactions (Table S1), initial protein concentrations (averages and coefficients of variation; Table S2), protein covariances (Table S3) and parameter values (Table S4) used in EARM1.3. (PDF) [file pcbi.1002482.s003.pdf]

SUPPLEMENTARY TABLES

Table S1. List of main reactions in EARM1.3

For all reactions, the general form is:

$$Species1 + Species\ 2 \underset{kr}{\overset{k}{\leftrightarrow}} Species\ 3 \xrightarrow{kc} Species\ 4 + Species\ 5$$

with parameters *k*, *kr* and *kc* identified below (see Tables S2 and S4 for parameter values).

Reaction	***Parameter names***
Ligand + Receptor ↔ Ligand:Receptor → Receptor*	*k(1), kr(1), kc(1)*
Receptor* + Flip ↔ Receptor*:Flip	*k(2), kr(2)*
Receptor* + Caspase-8 ↔ Receptor*:Caspase-8 → Receptor* + Caspase-8*	*k(3), kr(3), kc(3)*
Caspase-8* + Bar ↔ Caspase-8*:Bar	*k(4), kr(4)*
Caspase-8* + Caspase-3 ↔ Caspase-8*:Caspase-3 → Caspase-8* + Caspase-3*	*k(5), kr(5), kc(5)*
Caspase-3* + Caspase-6 ↔ Caspase-3*:Caspase-6 → Caspase-3* + Caspase-6*	*k(6), kr(6), kc(6)*
Caspase-6* + Caspase-8 ↔ Caspase-6*:Caspase-8 → Caspase-6* + Caspase-8*	*k(7), kr(7), kc(7)*
Caspase-3* + XIAP ↔ Caspase-3*:XIAP → Caspase-3*_Ub + XIAP	*k(8), kr(8), kc(8)*
Caspase-3* + PARP ↔ Caspase-3*:PARP → Caspase-3* + cPARP	*k(9), kr(9), kc(9)*
Caspase-8* + Bid ↔ Caspase-8*:Bid → Caspase-8* + tBid	*k(10), kr(10), kc(10)*
tBid + Mcl1 ↔ Bid:Mcl1	*k(11), kr(11)*
tBid + Bax ↔ tBid:Bax → tBid + Bax*	*k(12), kr(12), kc(12)*
Bax* ↔ Bax*_m	*k(13), kr(13)*
Bax*_m + Bcl2 ↔ Bax*_m:Bcl2	*k(14), kr(14)*
Bax*_m + Bax*_m ↔ Bax*2_m	*k(15), kr(15)*
Bax*2_m + Bcl2 ↔ Bax*2_m:Bcl2	*k(16), kr(16)*
Bax*2_m + Bax*2_m ↔ Bax*4_m	*k(17), kr(17)*
Bax*4_m + Bcl2 ↔ Bax*4_m:Bcl2	*k(18), kr(18)*
Bax*4_m + Pore ↔ Bax*4_m:Pore → Pore*	*k(19), kr(19), kc(19)*
Pore* + Cytoc_m ↔ Pore*:Cytoc_m → Pore* + Cytoc_r	*k(20), kr(20), kc(20)*
Pore* + Smac_m ↔ Pore*:Smac_m → Pore* + Smac_r	*k(21), kr(21), kc(21)*
Pore* → Pore	*kdeg(41)*
Cytoc_r ↔ Cytoc	*k(22), kr(22)*
Cytoc + Apaf ↔ Cytoc:Apaf → Cytoc + Apaf*	*k(23), kr(23), kc(23)*
Apaf* + C9 ↔ Apoptosome	*k(24), kr(24)*
Apoptosome + Caspase-3 ↔ Apoptosome:Caspase-3 → Apoptosome + Caspase-3*	*k(25), kr(25), kc(25)*
Smac_r ↔ Smac	*k(26), kr(26)*
Apoptosome + XIAP ↔ Apoptosome:XIAP	*k(27), kr(27)*
Smac + XIAP ↔ Smac:XIAP	*k(28), kr(28)*
Receptor* ↔ Ligand+ Receptor	*k(31), kr(31)*

Synthesis and degradation reactions

For all species (except Pore*, as noted in Table S1), the form is:

$$Species \underset{kdeg}{\overset{ks}{\leftrightarrow}} null$$

Where *ks* is the synthesis rate, *kdeg* is the degradation rate, as listed in Table S2.

Table S2. Initial species concentrations and synthesis/degradation rates for EARM1.3

	Model Species	HeLa base case initial concentration (IC) (molecules/cell)	Base case synthesis rate (*ks*, molecules/s)	Degradation rate (*kdeg*, 1/s)	Coefficient of variation
**1**	Ligand	3000†	0	2.9E-06	0
**2**	Receptor	1000	0.15*kdeg*IC	2.9E-06	0.25
**3**	Ligand:Receptor	0	0	2.9E-06	N/A
**4**	Receptor*	0	0	2.9E-06	N/A
**5**	Flip	2000	0.15*kdeg*IC	2.9E-06	0.25
**6**	Flip:Receptor*	0	0	2.9E-06	N/A
**7**	Caspase-8	10000	0.15*kdeg*IC	2.9E-06	0.25
**8**	Caspase-8:Receptor*	0	0	2.9E-06	N/A
**9**	Caspase-8*	0	0	2.9E-06	N/A
**10**	Bar	1000	0.15*kdeg*IC	2.9E-06	0.25
**11**	Caspase-8*:Bar	0	0	2.9E-06	N/A
**12**	Caspase-3	10000	0.15*kdeg*IC	2.9E-06	0.282
**13**	Caspase-8*:Caspase-3	0	0	2.9E-06	N/A
**14**	Caspase-3*	0	0	2.9E-06	N/A
**15**	Caspase-6	10000	0.15*kdeg*IC	2.9E-06	0.25
**16**	Caspase-3*:Caspase-6	0	0	2.9E-06	N/A
**17**	Caspase-6*	0	0	2.9E-06	N/A
**18**	Caspase-6*:Caspase-8	0	0	2.9E-06	N/A
**19**	XIAP	100000	0.15*kdeg*IC	2.9E-06	0.288
**20**	XIAP:Caspase-3*	0	0	2.9E-06	N/A
**21**	PARP	1000000	0.15*kdeg*IC	2.9E-06	0.25
**22**	Caspase-3*:PARP	0	0	2.9E-06	N/A
**23**	cPARP	0	0	2.9E-06	N/A
**24**	Bid	60000	0.15*kdeg*IC	2.9E-06	0.288
**25**	Caspase-8*:Bid	0	0	2.9E-06	N/A
**26**	tBid	0	0	2.9E-06	N/A
**27**	Mcl-1	20000	0.15*kdeg*IC	0.0001	0.25
**28**	tBid:Mcl-1	0	0	2.9E-06	N/A
**29**	Bax	80000	0.15*kdeg*IC	2.9E-06	0.271
**30**	tBid:Bax	0	0	2.9E-06	N/A
**31**	Bax*	0	0	2.9E-06	N/A
**32**	Bax*_m	0	0	2.9E-06	N/A
**33**	Bcl-2	30000	0.15*kdeg*IC	2.9E-06	0.294
**34**	Bax*_m:Bcl-2	0	0	2.9E-06	N/A
**35**	Bax*2_m	0	0	2.9E-06	N/A
**36**	Bax*2_m:Bcl-2	0	0	2.9E-06	N/A
**37**	Bax*4_m	0	0	2.9E-06	N/A
**38**	Bax*4_m:Bcl-2	0	0	2.9E-06	N/A
**39**	Pore	500000	0.15*kdeg*IC	2.9E-06	0.25
**40**	Bax*4:Pore	0	0	2.9E-06	N/A
**41**	Pore*	0	0	0.0001	N/A
**42**	CytoC_m	500000	0.15*kdeg*IC	2.9E-06	0.25
**43**	M*:CytoC_m	0	0	2.9E-06	N/A
**44**	CytoC_r	0	0	2.9E-06	N/A
**45**	Smac	100000	0.15*kdeg*IC	2.9E-06	0.25
**46**	M*:Smac	0	0	2.9E-06	N/A
**47**	Smac_r	0	0	2.9E-06	N/A
**48**	CytoC	0	0	2.9E-06	N/A
**49**	Apaf	100000	0.15*kdeg*IC	2.9E-06	0.25
**50**	Apaf:CytoC	0	0	2.9E-06	N/A
**51**	Apaf*	0	0	2.9E-06	N/A
**52**	Caspase-9	100000	0.15*kdeg*IC	2.9E-06	0.25
**53**	Apoptosome	0	0	2.9E-06	N/A
**54**	Apoptosome:Caspase-3	0	0	2.9E-06	N/A
**55**	Smac	0	0	2.9E-06	N/A
**56**	Apoptosome:XIAP	0	0	2.9E-06	N/A
**57**	Smac:XIAP	0	0	2.9E-06	N/A
**58**	Caspase-3*_Ub	0	0	0	N/A

† indicates a unitless constant.

Note 1: In all simulations the initial concentration of ligand was set to 3,000 which best mimics signaling dynamics observed in HeLa cells treated with 50 ng/ml of TRAIL.
Note 2: The synthesis rates are set for each protein as ks = 0.15*kdeg*IC such that they are only 15% of the value required for maintaining protein concentrations at steady state. This mimics the effects of treating HeLa cells with 2.5 ug/ml cycloheximide [1], as used in all experiments.

Table S3: Covariance matrix for Bax, Bcl-2, Bid, Caspase-3 and XIAP. The coefficients of correlation (R) which estimate the covariance after mean-centering and reducing the variables, where obtained from pairwise measurements of protein levels by flow cytometry and used to build a joint initial protein concentration distribution for these five proteins.

	**Bcl-2**	**Bid**	**Caspase-3**	**XIAP**	**Bax**
**Bcl-2**	1	0.477302	0.392112	0.673084	0.726968
**Bid**	0.477302	1	0.39913	0.37203	0.519564
**Caspase-3**	0.392112	0.39913	1	0.607546	0.672214
**XIAP**	0.673084	0.37203	0.607546	1	0.52748
**Bax**	0.726968	0.519564	0.672214	0.52748	1

Table S4: Kinetic rate parameters for EARM1.3.

**forward rates**	**1/(s*molec/cell)**	**reverse rates**	**1/s**	**catalytic rates**	**1/s**
k1	4.00E-07*	***kr1***	1.00E-06	***kc1***	1.00E-02
k2	1.00E-06	***kr2***	0.001		
k3	1.00E-07	***kr3***	0.001	***kc3***	1
k4	1.00E-06	***kr4***	0.001		
k5	1.00E-07	***kr5***	0.001	***kc5***	1
k6	1.00E-07	***kr6***	0.001	***kc6***	1
k7	1.00E-07	***kr7***	0.001	***kc7***	1
k8	2.00E-06	***kr8***	0.001	***kc8***	0.1
k9	1.00E-06	***kr9***	0.001	***kc9***	20
k10	1.00E-07	***kr10***	0.001	***kc10***	1
k11	1.00E-06	***kr11***	0.001		
k12	1.00E-07	***kr12***	0.001	***kc12***	1
k13	0.01	***kr13***	1		
k14	1.00E-06	***kr14***	0.001		
k15	1.00E-06	***kr15***	0.001		
k16	1.00E-06	***kr16***	0.001		
k17	1.00E-06	***kr17***	0.001		
k18	1.00E-06	***kr18***	0.001		
k19	1.00E-06	***kr19***	0.001	***kc19***	1
k20	2.00E-06	***kr20***	0.001	***kc20***	10
k21	2.00E-06	***kr21***	0.001	***kc21***	10
k22	1	***kr22***	0.01		
k23	5.00E-07	***kr23***	0.001	***kc23***	1
k24	5.00E-08	***kr24***	0.001		
k25	5.00E-09	***kr25***	0.001	***kc25***	1
k26	1	***kr26***	0.01		
k27	2.00E-06	***kr27***	0.001		
k28	7.00E-06	***kr28***	0.001		
k31	0.001	***kr31***	0		

* indicates units of 1/s

References

1. Ceccarini C, Eagle H (1976) Some paradoxical effects of inhibitors of protein synthesis on protein turnover in cultured human cells. In Vitro 12: 346-351.
